# Understanding the relationship between sequences and kinetics of DNA strand displacements

**DOI:** 10.1093/nar/gkae652

**Published:** 2024-07-30

**Authors:** Da Long, Peichen Shi, Xin Xu, Jiayi Ren, Yuqing Chen, Shihui Guo, Xinchang Wang, Xiaoyu Cao, Liulin Yang, Zhongqun Tian

**Affiliations:** State Key Laboratory of Physical Chemistry of Solid Surface, Key Laboratory of Chemical Biology of Fujian Province, Collaborative Innovation Center of Chemistry for Energy Materials (iChEM), Innovation Laboratory for Sciences and Technologies of Energy Materials of Fujian Province (IKKEM), College of Chemistry and Chemical Engineering, Xiamen University, Xiamen 361005, PR China; State Key Laboratory of Physical Chemistry of Solid Surface, Key Laboratory of Chemical Biology of Fujian Province, Collaborative Innovation Center of Chemistry for Energy Materials (iChEM), Innovation Laboratory for Sciences and Technologies of Energy Materials of Fujian Province (IKKEM), College of Chemistry and Chemical Engineering, Xiamen University, Xiamen 361005, PR China; State Key Laboratory of Physical Chemistry of Solid Surface, Key Laboratory of Chemical Biology of Fujian Province, Collaborative Innovation Center of Chemistry for Energy Materials (iChEM), Innovation Laboratory for Sciences and Technologies of Energy Materials of Fujian Province (IKKEM), College of Chemistry and Chemical Engineering, Xiamen University, Xiamen 361005, PR China; State Key Laboratory of Physical Chemistry of Solid Surface, Key Laboratory of Chemical Biology of Fujian Province, Collaborative Innovation Center of Chemistry for Energy Materials (iChEM), Innovation Laboratory for Sciences and Technologies of Energy Materials of Fujian Province (IKKEM), College of Chemistry and Chemical Engineering, Xiamen University, Xiamen 361005, PR China; State Key Laboratory of Physical Chemistry of Solid Surface, Key Laboratory of Chemical Biology of Fujian Province, Collaborative Innovation Center of Chemistry for Energy Materials (iChEM), Innovation Laboratory for Sciences and Technologies of Energy Materials of Fujian Province (IKKEM), College of Chemistry and Chemical Engineering, Xiamen University, Xiamen 361005, PR China; School of Informatics, Xiamen University, Xiamen 361005, PR China; School of Electronic Science and Engineering (National Model Microelectronics College), Xiamen University, Xiamen 361005, PR China; State Key Laboratory of Physical Chemistry of Solid Surface, Key Laboratory of Chemical Biology of Fujian Province, Collaborative Innovation Center of Chemistry for Energy Materials (iChEM), Innovation Laboratory for Sciences and Technologies of Energy Materials of Fujian Province (IKKEM), College of Chemistry and Chemical Engineering, Xiamen University, Xiamen 361005, PR China; State Key Laboratory of Physical Chemistry of Solid Surface, Key Laboratory of Chemical Biology of Fujian Province, Collaborative Innovation Center of Chemistry for Energy Materials (iChEM), Innovation Laboratory for Sciences and Technologies of Energy Materials of Fujian Province (IKKEM), College of Chemistry and Chemical Engineering, Xiamen University, Xiamen 361005, PR China; State Key Laboratory of Physical Chemistry of Solid Surface, Key Laboratory of Chemical Biology of Fujian Province, Collaborative Innovation Center of Chemistry for Energy Materials (iChEM), Innovation Laboratory for Sciences and Technologies of Energy Materials of Fujian Province (IKKEM), College of Chemistry and Chemical Engineering, Xiamen University, Xiamen 361005, PR China

## Abstract

Precisely modulating the kinetics of toehold-mediated DNA strand displacements (TMSD) is essential for its application in DNA nanotechnology. The sequence in the toehold region significantly influences the kinetics of TMSD. However, due to the large sample space resulting from various arrangements of base sequences and the resulted complex secondary structures, such a correlation is not intuitive. Herein, machine learning was employed to reveal the relationship between the kinetics of TMSD and the toehold sequence as well as the correlated secondary structure of invader strands. Key factors that influence the rate constant of TMSD were identified, such as the number of free hydrogen bonding sites in the invader, the number of free bases in the toehold, and the number of hydrogen bonds in intermediates. Moreover, a predictive model was constructed, which successfully achieved semi-quantitative prediction of rate constants of TMSD even with subtle distinctions in toehold sequence.

## Graphical Abstract

**Graphical Abstract:**
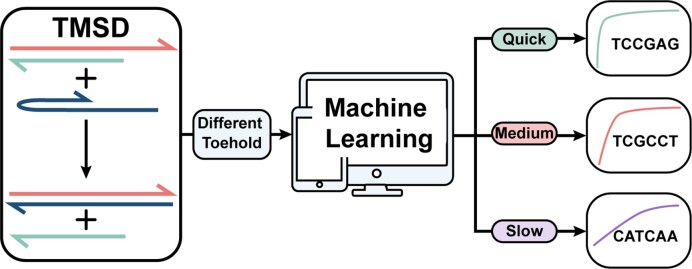

## Introduction

DNA (1) as an important building block has been widely employed to construct various functional nanodevices (2–6) and stimuli-responsive reaction networks (7,8), benefiting from its precise and programmable assembly (9–13). Currently, DNA-based nanotechnology has found applications in many fields such as drug delivery (14,15), bio-imaging (16), molecular computing (17–20), and micro/nano-robotics (21,22).

**Figure 1.**
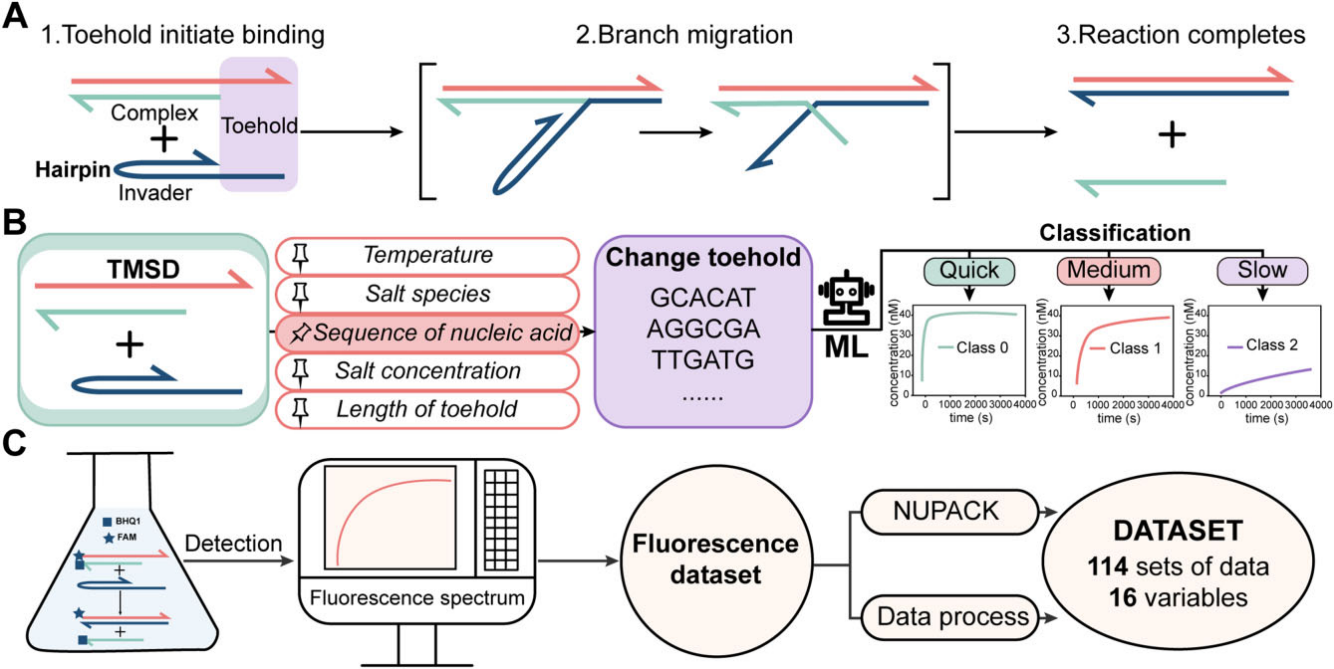
This figure primarily introduces the study of the DNA strand displacement mechanism, including its fundamental processes, the use of machine learning to analyze the relationship between DNA sequences and displacement rates, and the sources of the dataset. (**A**) Schematic representation of the DNA strand displacement, featuring the invader with a hairpin structure. The entire process involves three steps – toehold binding, branch migration, and the displacement of the invader strand. (**B**) Machine learning was employed to study the relationship between DNA sequences and rate constants of strand displacements by altering the sequence in the toehold, while other factors remained constant. (**C**) Source of the dataset: fluorescence monitoring of strand displacements provides kinetic data, while binding probabilities obtained from NUPACK collectively constitute the entire dataset.

The toehold-mediated DNA strand displacement (TMSD) is a fundamental and most widely used assembly in DNA nanotechnology (7,23,24). Precisely controlling the kinetics of DNA strand displacements helps us construct intricate and precise DNA nanodevices. According to the mechanism of TMSD, the invading ssDNA and the substrate dsDNA form a three-stranded intermediate mediated by toeholds, which is generally assumed as the rate-limiting step, and then the strand replacement is completed through branch migration (24). Therefore, besides external factors like temperature, pH (25), salt (26), solvent (27) and luminescent labels (28), the rate of a DNA strand displacement is intricately connected to the number, types and sequences of bases on the toehold (29–32). As the number of bases increases from zero to six in the toehold, the rate constants of displacements can increase by six orders of magnitude. Further increasing the number of bases in the toehold has little effect on the rate constants (33). Moreover, even equal-length toeholds with different sequences can result in rate constants differing by three to four orders of magnitude (23). Since the different number of hydrogen bonds in A/T and G/C base pairs, the proportion of the two types of base pairs formed in the toehold region between the invading and substrate strands can affect the stability of the intermediate, and thereby affect the rate of strand displacement. However, considering only the primary structure of the toehold segment is not enough to explain the difference of several orders of magnitude in the displacement rate. As a typical macromolecule with multiple binding sites, single-strand DNA may form secondary structures such as hairpins through intramolecular base pairing (Figure 1A), which can hinder the progress of strand displacement and significantly affect the rate of displacement. Even if the sequence of the branch migration region is fixed, changing the toehold primary sequence can still lead to complex chain conformations through intramolecular interactions, thereby affecting the potential energy surface and rate constants of strand displacement. Therefore, to understand the kinetics of DNA strand displacement from a physicochemical perspective, it is necessary to establish the correlation from primary sequence and secondary conformation to activation energy and rate constants. However, due to the large sample space resulting from various arrangements of base sequences, such a correlation is not intuitive.

In recent years, the rapid advancement of machine learning has provided us with powerful tools to tackle complex problems (34,35). Previous reports have employed machine learning methods to mine sequence information. An algorithm called weighted neighbour voting has been used to predict the rate constants of DNA hybridization reactions (36). This model offers an effective approach for probe design in genomics. A decision tree model was constructed to estimate the rate constants of strand displacements based on external toehold protection strategy (ETP) to mitigate the interference from other strands in DNA pools (37). This offers inspiration for designing sequences with ETP in complex systems. A deep learning model which included a bidirectional recurrent neural network was also used to predict the sequencing depth of Next-Generation Sequencing (NGS) from DNA probe sequences and DNA interaction kinetics rate constants (38).

In this work, the relationship between sequences and the rate constants of strand displacements has been investigated assisted by machine learning (Figure 1B). Using the random forest algorithm, the key factors that influence the rate constants of strand displacements have been identified, including the number of free hydrogen bonding sites in the invader, the number of free bases in the toehold, and the number of hydrogen bonds in intermediates. The correlation from primary sequence and secondary conformation to activation energy and rate constant has been discussed based on these key variables. Simultaneously, a predictive model using classical machine learning methods has been constructed, which achieved semi-quantitative prediction of rate constants of DNA strand displacements.

## Materials and methods

### Material

All DNA oligonucleotides were sourced from Sangon Biotechnology Co., Ltd (Shanghai, China), as detailed in Supplementary Information Table S1. Oligonucleotides labelled with fluorescent dyes or quenchers were purified using high-performance liquid chromatography (HPLC), while unmodified oligonucleotides were purified using ULTRAPAGE. A buffer solution of 1 M Tris–HCl was purchased from Shanghai Acmec Biochemical Co., Ltd (Shanghai, China). Ethylenediamine tetra-acetic acid disodium salt dihydrate and sodium hydroxide were purchased from Sinopharm Chemical Reagent Co., Ltd (Shanghai, China).

### Methods

#### Design of DNA strands

The substrate strands (F) were labelled with the fluorescent dye 5(6)-carboxyfluorescein (5(6)-FAM) at their 5′ terminus, while the 3′ terminus of the incumbent strands (Q) was marked with the carboxylic acid quencher (BHQ1). Initially, the fluorescence of the dye was quenched due to the binding of the QF strand. Upon strand displacement, as the invader strand (I) replaced the incumbent strand (Q), the 5′ end of the substrate strand (F) annealed to the 3′ end of the invader strand (I), leading to the restoration of fluorescence. In the strand design, both the substrate and invader strands consisted of 22 bases, whereas the incumbent strands were composed of 16 bases. The toehold sequence was varied randomly, while the branch migration region remained constant. All sequences are available in Supplementary Information Table S1.

#### Buffer preparation

To prepare a 10 mM Tris–HCl buffer, 12 mmol MgCl_2_·6H_2_O, 10 ml of 1 M Tris–HCl and 1 mmol Na_2_EDTA·2H_2_O were mixed. Then the mixture was diluted with 990 ml ultrapure water. Finally, the pH of the solution was adjusted to 7.50 using a 3 M NaOH solution.

#### Calibration of the concentration of oligonucleotide acids

The oligonucleotide acid strands in each centrifuge tube were present at a concentration of 1 nmol. The tube was centrifuged for 60 s at 1698 × g to collect the DNA lyophilized powder at the bottom of the tube. Next, 100 μl of buffer salt was added, and the solution was vigorously vortexed to ensure complete dissolution of the sample. The concentration of the sample was determined using a micro-UV spectrophotometer, and then diluted to 1 μM with buffer salt to obtain the stock solution.

#### Preparation of the sample solution

The TMSD involves three oligonucleotide strands: the substrate strand (F), the incumbent strand (Q), and the invader strand (I). Each TMSD set was prepared as follows: a 1 μM mixed stock solution of Q and F strands was first diluted to a 100 nM concentration in a 5 ml solution, while a separate 1 μM stock solution of I strands was similarly diluted to a 100 nM in a 5 ml solution. In addition, a 2 ml solution containing a 100 nM mixed combination of I and F strands was prepared to establish a standard curve correlating fluorescence intensity with the concentration of IF strand.

#### Annealing treatment

The mixed solutions of I and F strands, as well as the mixed solutions of Q and F strands, were subjected to heating in a water bath at 90°C for 15 min, followed by transfer to another water bath at 50°C for 5 min. Subsequently, the samples were removed and allowed to cool naturally to room temperature before use. The solution of strand I was not annealed.

#### Collection of kinetic data of TMSD

The progression of TMSD was monitored using a fluorescent probe. The experiment was set with a fluorescence excitation wavelength of 480 nm, an excitation slit width of 5 nm, an emission wavelength of 517 nm, and an emission slit width of 10 nm. (i) Standard curve establishment: A series of dilutions of 100 nM IF solution were prepared using buffer solution to obtain concentrations of 75, 50, 25, 10 nM. The fluorescence intensity at these five concentrations was measured to establish the correlation between fluorescence intensity and concentration. (ii) Background scanning: A mixture of 1 ml of 100 nM QF solution and an equal volume of Tris–HCl buffer solution was prepared, and its fluorescence intensity was measured as the background value. (iii) Determined the kinetics of strand displacements: 1 ml solution of 100 nM I strand was placed in a 1 cm optical path length quartz cuvette (containing a magnetic stir bar). An equal volume of QF strand solution with the same concentration was injected, and the timer was started. After sealing the cuvette, it was placed in the fluorescence cell and continuously monitored until the reaction was complete. The detailed processing procedure can be found in Supplementary Information Figure S1. Each sequence group needs to undergo the above three steps. To ensure the reliability and reproducibility of kinetics data under experimental conditions, we selected 80 group samples, and each group sample underwent three parallel experiments. All the raw kinetic data can be found in Supporting Information Figure S2.

#### Variable temperature kinetics experiment

The fluorescence instrument was combined with the constant temperature device. The temperature was set to 15°C. After the temperature was stable, a volume of 1 ml of QF solution was mixed with a volume of 1 ml of strand I solution, then fluorescence kinetics monitoring was performed. The reaction time was set to 2 h, and other conditions were consistent with the above experimental conditions. The temperature was set at 20°C, 25°C, 30°C and the above steps were repeated to obtain four sets of data for each sample. All experimental data of temperature-variable kinetics are provided in the Supporting Information Figure S4.

## Results

### Identify key variables that correlate the sequences with the rate constants of TMSD

**Figure 2.**
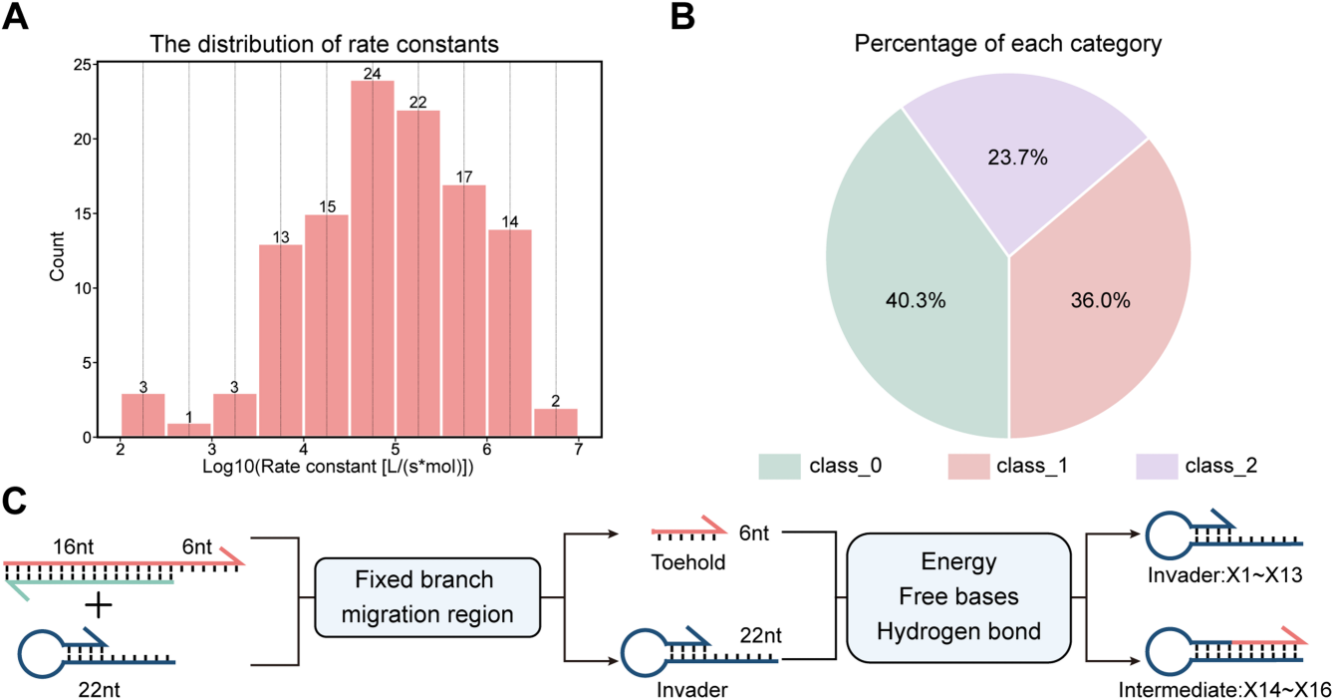
This figure provides insights into the experimental data analysis, covering the distribution of rate constants, the proportions of different rate constant classes, and the construction of variables to describe the DNA strand interactions. (**A**) The distribution of rate constants from the 114 sets of experiments. The rate constants of samples in the dataset vary within five orders of magnitude. (**B**) Proportions of three classes of rate constants in the dataset. (**C**) The construction of variables. In 114 sets of samples, 16 nucleotides in the branch migration region of the QF strands were all complexed, that means this region of DNA strands were in the same initial state. Therefore, only the interactions among six nucleotides in the toehold region of the substrate strand and the entire 22 nucleotides in the invader strand were considered for constructing variables. From the perspective of interaction energy, free bases, and the number of hydrogen bonds, 13 variables were constructed to describe the primary and secondary structures of invader strands and three variables to describe the formation of intermediates (details in Supplementary Information Table S2).

The invaders were designed by randomly generating six bases in the toehold, while 16 bases in the branch migration region were fixed. This design considered two factors: (i) the binding of toeholds is generally regarded as the rate-determining step in strand displacement, and altering the sequence in this region can lead to significant rate changes; (ii) although the sequence in the branch migration region was fixed, changing the sequence in the toehold region can also alter the secondary structure formed by interactions between these two regions. To obtain standardized data, purification methods for all DNA strands, experimental procedures for fluorescence kinetics, and data processing methods were kept consistent. The 5′ end of the substrate strand is labelled with a FAM fluorescent group and binds to the incumbent strand labelled with BHQ1 at the 3′ end to form a double-stranded complex. Subsequently, the invader solution was mixed with the complex solution. Kinetic data was fitted using the classic second-order kinetic equation (see Supplementary Information, Section II), the distribution of rate constants can be found in Figure 2A. A dataset comprising 114 sets of sequences matched with their corresponding strand displacement rate constants were finally collected (Figure 1C).

To construct the relationship between sequences and strand displacement rate constants using machine learning, the sequence information was encoded. 16 variables were constructed by considering interactions among all the free bases in the system, including the bases of the invader and the bases of the toehold of double-stranded complex (see Supplementary Information, Figure S3). Among them, 13 variables were correlated with the primary and secondary structure of invader strands. The formation of hairpin-like secondary structures of an invader strand can be associated by the paring probability of each base (data available from the online NUPACK (39) tool), the number of hydrogen bonds formed in the minimum free energy structure of invaders, the number of free hydrogen bonding sites in the toehold, and so on. It is proposed that the TMSD should be mediated by the formation of a three-chain intermediates through the complexation of toeholds between the invader and substrate strands. Therefore, another three variables were constructed to describe the intermediates (Figure 2C). To simplify the investigation, the rate constants of TMSD were classified into three classes based on the time required to equilibrium: <1000 s as class 0, between 1000 and 3600 s as class 1, and >3600 s as class 2, the proportions of three classes of data is shown in Figure 2B. Machine learning was employed to aid in exploring how DNA sequences significantly affect strand displacement kinetics.

**Figure 3.**
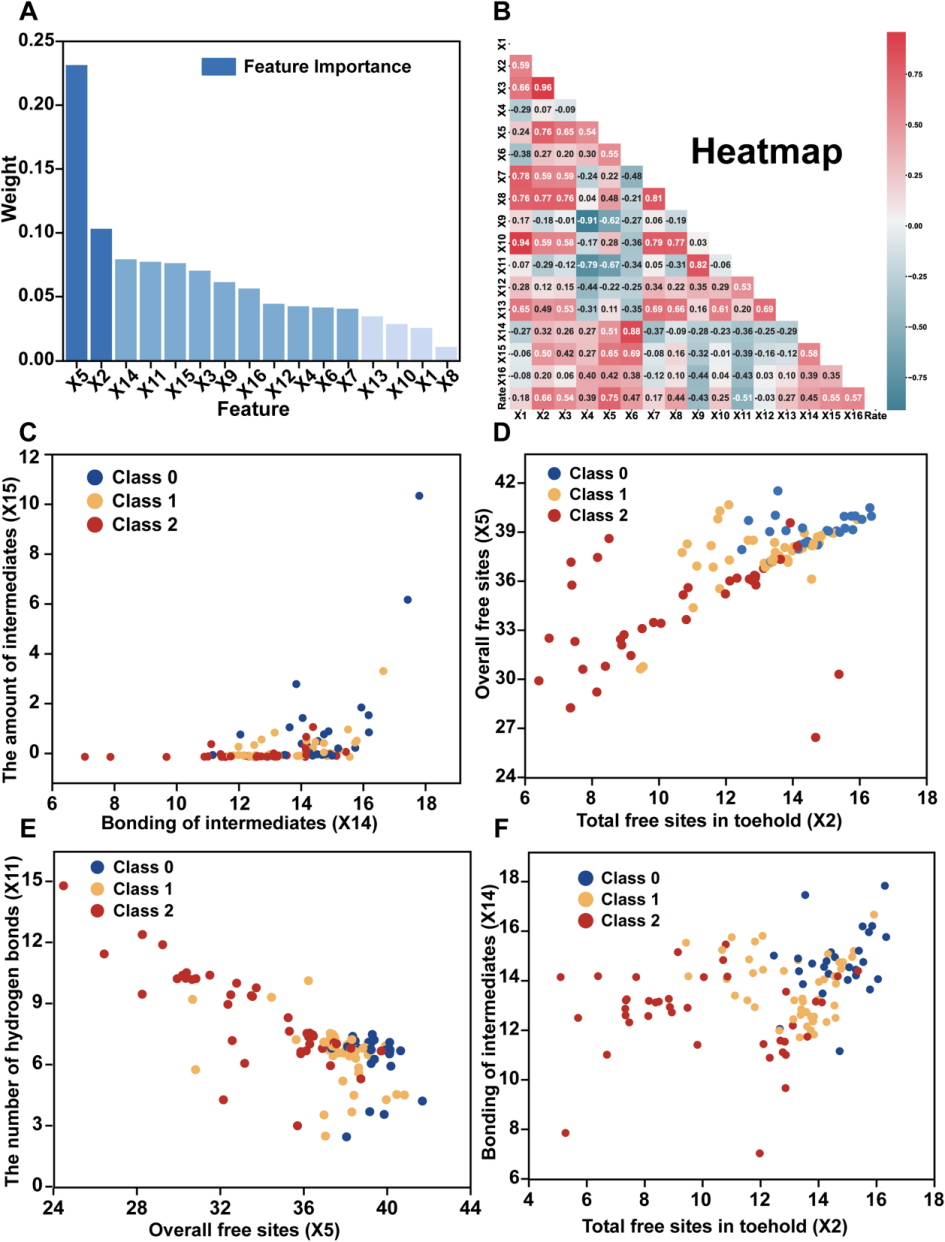
The comprehensive analysis depicted here encompasses variable importance ranking, correlation heatmap, and sample distribution related to key variables. (**A**) Importance ranking of variables: weight of each variable based on the Random Forest algorithm. (**B**) Correlation heatmap: correlations between pairs of variables based on Pearson coefficients. (**C**) The distribution of samples is related to the number of hydrogen bonds involved in the formation of intermediates (X14) and the amount of intermediates (X15). (**D**) Sample distribution connected to the number of free hydrogen bonding sites in the toehold (X2) and the number of free hydrogen bonding sites along the entire invader (X5). (**E**) Sample distribution associated with variables X5 and X11. (**F**) Sample distribution tied to variables X2 and X14.

The Random Forest algorithm was employed to prioritize the 16 variables (Figure 3A). The most important variable is the number of free hydrogen bonding sites of all bases (X5) on the invader. This variable is highly correlated to the intra-chain folding of invader chains (for instance, hairpin structures) that occupy or protect a part of bases. By correlation analysis (Figure 3B), the rate constants show a positive correlation with the number of free hydrogen bonding sites along the entire invader (X5), the number of free hydrogen bonding sites in the toehold (X2), the number of hydrogen bonds involved in the formation of intermediates (X14), and the content of intermediates (X15). In contrast, the rate constants have negative correction with the number of hydrogen bond pairs in the branch migration region (X9), the number of hydrogen bonds in the minimum free energy structure of the invader (X11), and the number of bases in the loop (X12).

Faster strand displacements can be achieved when more hydrogen bonds are involved in the formation of ternary-complex intermediates (40). However, the rate constants do not exhibit a strong correlation with the amount of the hypothetical intermediates (Figure 3C). Both the number of free hydrogen bonding sites on the invader and the number of hydrogen bonding sites in the toehold significantly influence the rates of strand displacements, showing a positive correlation (Figure 3D). Moreover, a lower number of base pairs in the minimum free energy structure results in more hydrogen bonding sites on the invader strands and leads to faster strand displacements (Figure 3E). In addition, a higher number of hydrogen bonding sites in the toehold results in a faster rate of the strand displacement (Figure 3F). The higher the number of hydrogen bonds involved in the formation of intermediates, the more stable the intermediates will be. As shown in Figure 3C and F, the values of the X14 are higher for almost all fast displacements than for medium and slow ones. Therefore, the X14 offers a precise representation of how intermediate stability influences the rate constants of strand displacements.

### Establishing models for estimating the rate constants of TMSD

**Figure 4.**
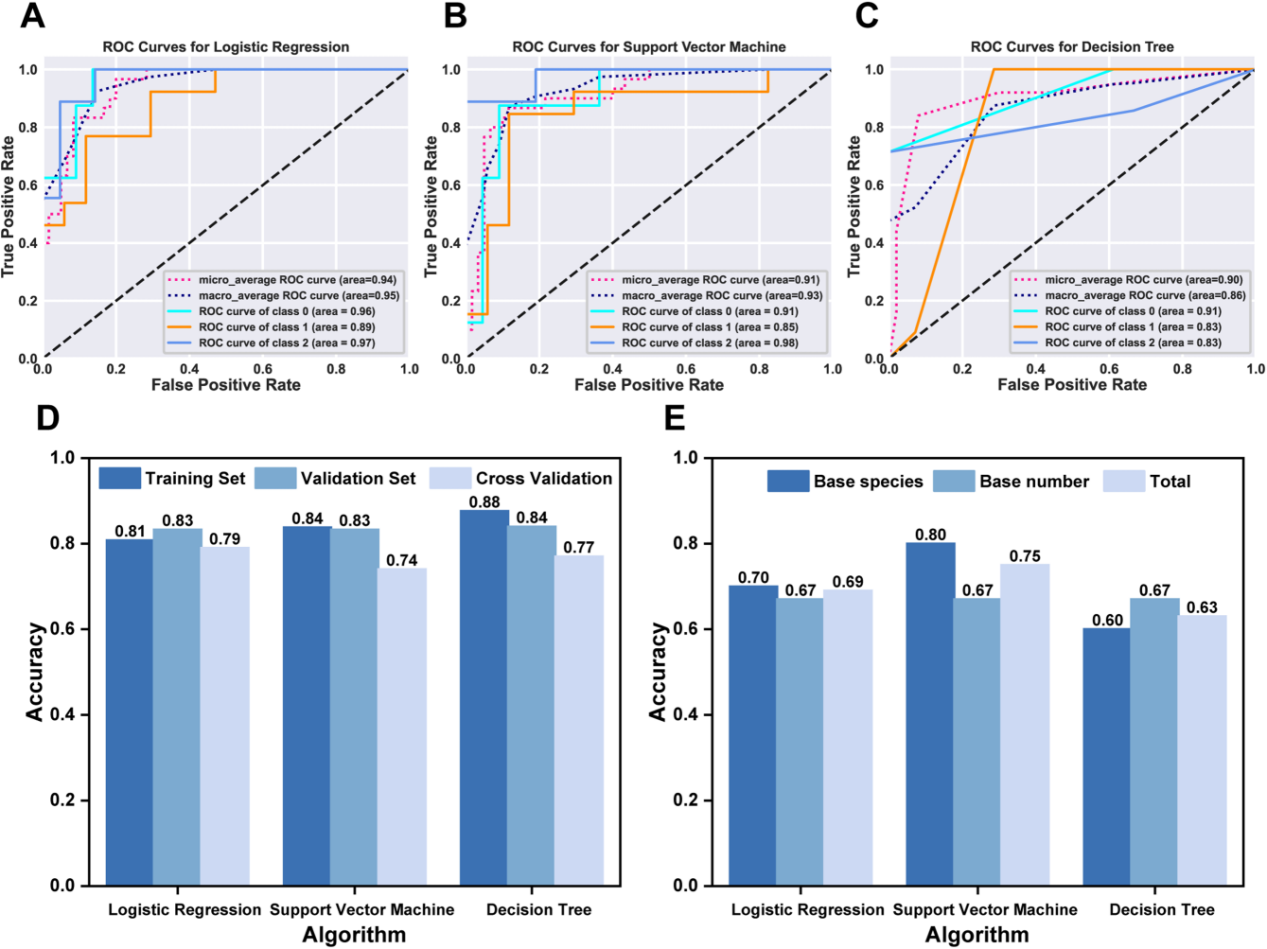
The evaluation metrics of the model and the test results. (**A–C**) ROC curves: demonstrating the performance of Logistic Regression, Support Vector Machine and Decision Tree models. The ROC curve plots true positive *vs*. false positive rates for a classifier. A higher area under the curve (AUC) indicates better performance, with 1 being perfect and 0.5 being random guessing. (**D**) Model accuracy on the training set, validation set, and cross-validation. Details refer to Supplementary Table S4-S6 in Supplementary Information. (**E**) Model accuracy on the test set. The detailed data can be found in Supplementary Information Table S7.

Three traditional machine learning models (logistic regression, support vector machine, decision tree) were employed to build the relationship between DNA sequences and rate constants. The selection of these lightweight classification models considers the relatively small amount of data for the entire prediction task, and the fact that the combination with hand-crafted features provides better interpretability. The specific introduction of the algorithm and its mathematical logic are provided in the Supplementary Information (Section V). The ROC curve is a key metric for evaluating the performance of classification models, measured by calculating the area under the curve (AUC). Ideally, a higher AUC closer to 1 indicates better performance, while 0.5 is equivalent to random guessing. In Figure 4A–C, we observed that the area under the ROC curve for each class of the three models exceeded 0.83, and both the micro-average and macro-average AUCs also surpassed 0.85, indicating excellent classification performance of these models on the dataset. To further verify the performance of the models, we divided the data into training and validation sets and found that the accuracy of all three models exceeded 0.8. Moreover, to eliminate the impact of data order, we also employed cross-validation, and the results indicated that the accuracy of the models was still maintained at around 0.75 (Figure 4D). To assess the predictive ability of the model beyond the dataset, a test set comprising 16 samples was established (Figure 4E). Among them, 10 samples had alterations of base types within the branch migration region. The remaining 6 samples varied the number of bases on the substrate strand within the same region, with a total count ranging from 20 to 23. Because of the efficient handling of high-dimensional data by kernel functions, the support vector machine performs the best. For the 10 samples with varied base types, the model can predict the rate constants with high accuracy. For the rest 6 samples with different number of bases, the accuracy slightly decreased. This is due to all DNA strands in the training set having a base count of 22 nt, failing to cover samples with different base counts. While the decision tree algorithm demonstrated outstanding performance on both the training and validation sets, its accuracy on the test set was lower, possibly because the overall dataset is relatively small, and the data distribution in the test set differs from that used for model training. Overall, the three models excel in handling small samples, especially when using Support Vector Machine, which can distinguish the rate constants of different base types of samples with high precision. More details on the construction of models can be found in Supplementary Information Figure S5.

### Assessing the rate of TMSD from sequences assisted by the established models

For DNA strands with significant differences in toehold sequences, these models can accurately and quickly judge the magnitude of their displacement rate constants. Furthermore, the multiple key variables provided by the models are helpful for us to understand deeply how the sequences and secondary structures affect the rate constants of TMSD.

**Figure 5.**
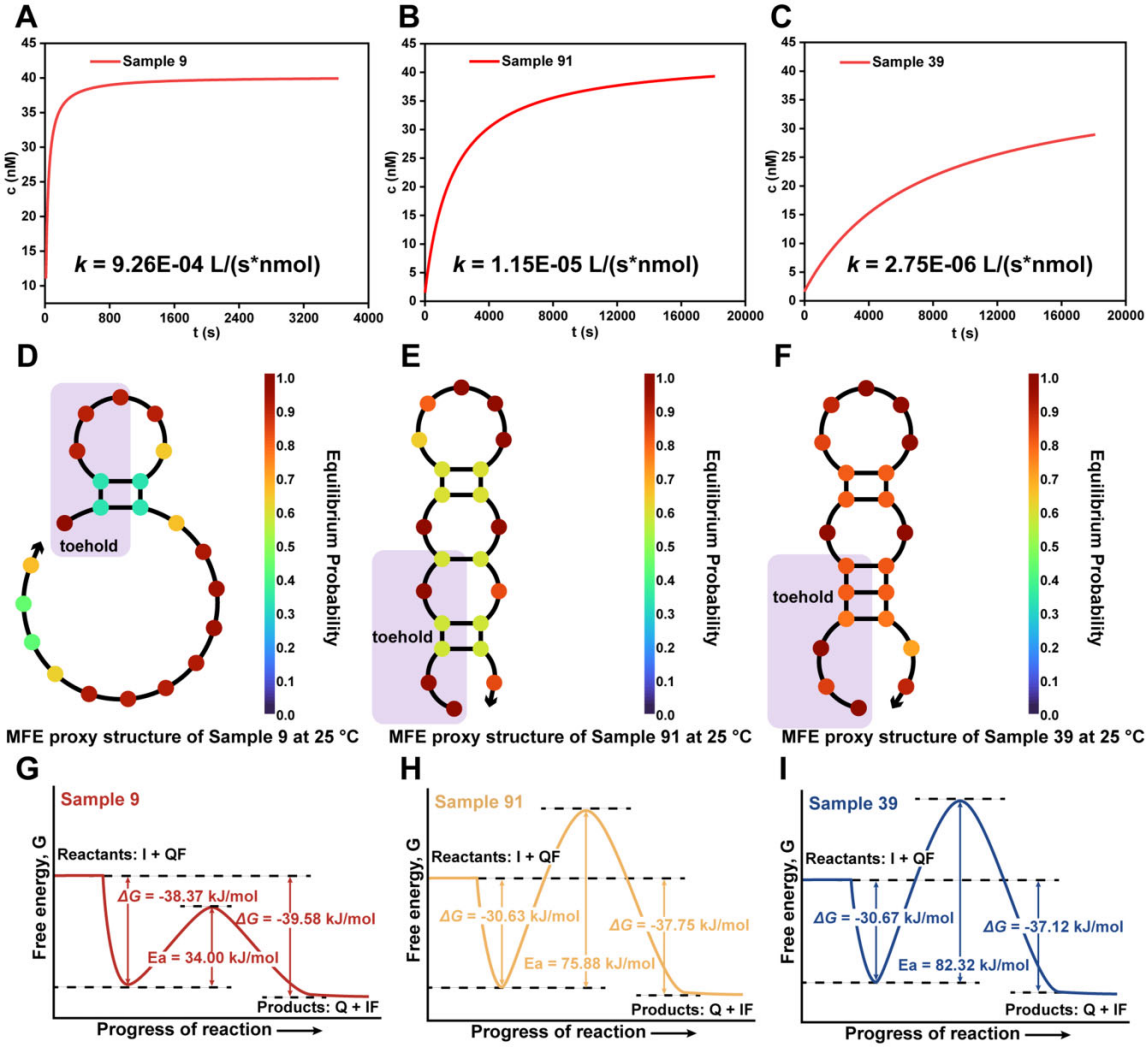
Understanding the relationship between key variables and kinetics based on three representative samples. (**A–C**) Kinetic fitting curves of strand displacements for Sample **9**, Sample **91**, and Sample **39**. (**D–F**) Schematic representation of the minimum free energy (MFE) structures for the invading strands of Sample **9**, Sample **91**, and Sample **39** (accessible through NUPACK). (**G–I**) Energy curves of strand displacements for Sample **9**, Sample **91**, and Sample **39**. The detailed calculation steps are in Supplementary Table S3 of the Supplementary Information.

As a representative, samples **9**, **39** and **91** were selected from the dataset (see Supplementary Information, Table S8). The determined rate constants of the three samples followed an order of sample **9** > sample **91** > sample **39** (Figure 5A–C). From the point of view of energy barrier (Figure 5G-I), the determined apparent activation energies of three samples were in an order of sample **9** < sample **91** < sample **39**, which explains the differences of rate constants of these samples. The key variables as well as the established models further help understand the orders of the energy barriers and rate constants of these three samples from the molecular structural perspective. According to the minimum free energy (MFE) structures of invader strands (Figure 5D-F), the number of unpaired bases in the toeholds followed the order of sample **91** =sample **39**< sample **9**. This suggested that sample **9** should has the highest rate constant of displacement. It was found that sample **9** (with a hydrogen bond count of 3.78) formed fewer and less stable intramolecular hydrogen bonds (X11) compared to sample **91** (with a hydrogen bond count of 10.07) in the invader. As a result, the intramolecular folding structure of sample **9** could be more easily disrupted, and the overall number of unpaired hydrogen bonding sites in the toehold (X2) was higher (12.69 for the former and 8.83 for the latter), leading to a higher strand displacement rate. The MFE structures of sample **91** and **39** both had three bases occupied in the toehold, and an equal number of base pairs throughout the whole strand. However, the three paired bases in the toehold of sample **39** were continuous without any interval base, while paired toehold bases of sample **91** were separated by a free base. This should result in a stronger synergistic effect of the three base pairs in sample **39** than that of sample **91**, suggesting higher hydrogen bonding pairing probabilities of the bases in sample **39**. Indeed, the three toehold bases in sample **39** all had a pairing probability of around 0.8, of which in sample **91** was around 0.6. This explains why the energy barrier of sample **39** was higher than sample **91**, and a significantly lower rate constant as a result. This example demonstrates that the key variables and the machine learning models are capable to describe the energy barrier of TMSD, quickly estimate the rate of strand displacement, and understand the relationship between molecular structures and kinetics, without the need for time-consuming temperature-dependent kinetic experiments to determine the apparent activation energy.

**Figure 6.**
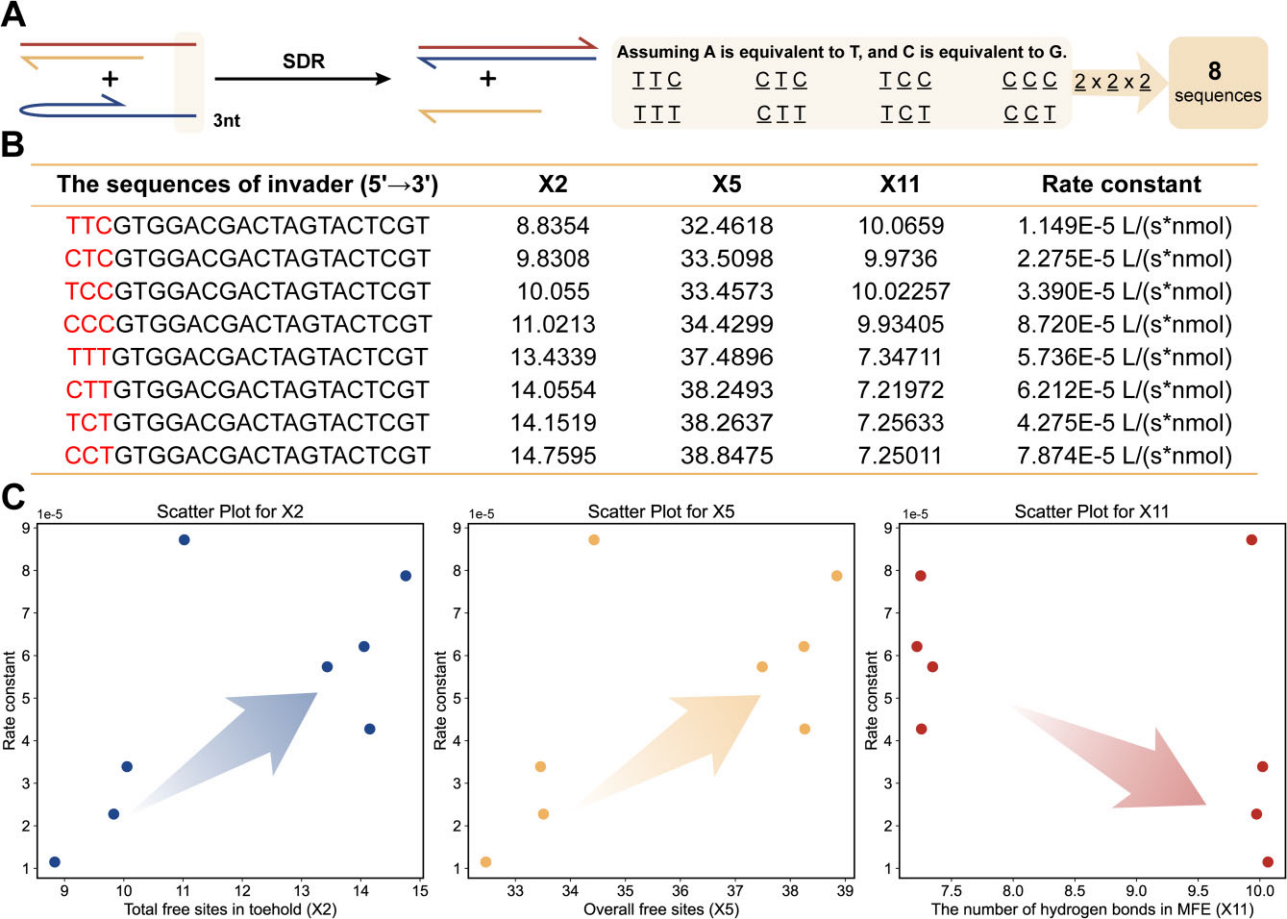
The impact of subtle sequence variations on strand displacement kinetics. (**A**) Schematic diagram of the design approach for nucleic acid sequences with subtle differences. (**B**) In the presented table, each column encompasses the following data points: the sequence of the invader, the variables X2 (Total free sites in toehold), X5 (overall free sites) and X11 (The number of hydrogen bonds in MFE), and the experimentally determined rate constants. (**C**) Scatter plots for the three key variables X2, X5 and X11 against the rate constants.

For samples in the same class, the rate constants are close to each other, and it is challenging to sort them manually. According to the key parameters (X2/X5/X11) proposed in these models, the magnitude of rate constants with small gaps can be well sorted, even with subtle differences in sequences. Changing the types of the first three bases in the toehold region can lead to variations in the rate constant just within an order of magnitude (Figure 6A). An order can be given based on the values of the variables X2, X5 and X11, which basically matched with the measured order of rate constants (Figure 6B). The general rule should be the more available hydrogen bonding sites in the -toehold (X2) as well as the entire invader (X5), and the fewer hydrogen bonding sites occupied by intra-strand pairing (X11), the higher the rate constants of strand displacements (Figure 6C).

## Discussion

According to our study, to achieve high rates for a simple TMSD system, the design of DNA strands can follow three rules. (i) Maximize the number of bases in the toehold. (ii) Increase the proportion of guanines and cytosines to provide more hydrogen bonding sites. (iii) Minimize the intrachain base pairs and avoid the occupation of toehold bases in the invader strand.

Complex TMSD systems involve multiple strands simultaneously, an invader may easily engage in partial pairing with non-target segments from other strands, thereby disrupting the targeted strand displacement. Therefore, the best input sequences of invader strands may not be those without any intrachain base pairing, but instead sequences with hairpin structure to prevent interference. This can be referred to the internal toehold protection (ITP) strategy (37). It not only enhances the yield of DNA walkers (41) but also prevents crosstalk in logic circuits (42). Thereby, to achieve high rates with minimized interference for complex TMSD circuits, the following rules are suggested. (i) In a multi-step cascade reaction, the toehold can be concealed within a hairpin structure to prevent interference, while being timely released to ensure efficient overall reactions. (ii) If internal protection needs to occupy bases in toeholds, ensure that the overall number of hydrogen bonds formed by the invader remains as low as possible. However, this approach may result in a reduction in the efficiency of the protection. (iii) Increase the proportion of unoccupied guanines and cytosines, especially in the toehold. This facilitates the association and dissociation processes of strand displacements.

## Conclusions

In conclusion, this work has revealed how the primary sequence and secondary structures of DNA strands affect rates of DNA strand displacements. Key factors were identified, and classification models based on these factors were established assisted by machine learning. These models provide a well estimation of the rate constants for the TMSD. Moreover, rules are proposed for designing controllable TMSD systems. The assembly of DNA is a representative assembly system that involves synergistic effects among multiple binding sites and multiple non-covalent interactions. It is anticipated that the interpretable machine learning can serve as a powerful tool to uncover the black boxes of kinetics of complex molecular assembly systems, and facilitate the construction and regulation of such systems.

## Supplementary Material

gkae652_Supplemental_Files

## Data Availability

The data that support the findings of this study are contained within the article and the supplementary information. All source data generated for this study are available from the corresponding author (Liulin Yang; llyang@xmu.edu.cn) upon reasonable request.
