# Supplementary material for: Understanding the relationship between sequences and kinetics of DNA strand displacements
Source: Nucleic Acids Res. 2024 Jul 30;52(16):9407–16. doi: 10.1093/nar/gkae652 (PMC11381357; doi:10.1093/nar/gkae652)
Supplement: gkae652_Supplemental_Files [file gkae652_supplemental_files.zip › SupportInformation_20240723.docx]

**Supplemental Information**

**Understanding** **the Relationship between Sequence and**

**Kinetics of DNA Strand Displacements**

Da Long,^1^ Peichen Shi,^1^ Xin Xu,^1^ Jiayi Ren,^1^ Yuqing Chen,^1^ Shihui Guo,^3^ Xinchang Wang,^2^ Xiaoyu Cao,*^1^ Liulin Yang,*^1^ and Zhongqun Tian^1^

^1^ State Key Laboratory of Physical Chemistry of Solid Surface, Key Laboratory of Chemical Biology of Fujian Province, Collaborative Innovation Center of Chemistry for Energy Materials (iChEM), Innovation Laboratory for Sciences and Technologies of Energy Materials of Fujian Province (IKKEM), College of Chemistry and Chemical Engineering, Xiamen University, Xiamen 361005, P. R. China, E-mail: [llyang@xmu.edu.cn](mailto:llyang@xmu.edu.cn), xcao@xmu.edu.cn

^2^ School of Electronic Science and Engineering (National Model Microelectronics College), Xiamen University, Xiamen 361005, P. R. China

^3^ School of Informatics, Xiamen University, Xiamen 361005, P. R. China

**Table of Contents:**

1. **Instruments**
2. **Experimental Data and Model Fitting**
3. **Feature Extraction and Feature List**
4. **Temperature Dependent Kinetics Experiments**
5. **Machine Learning and Dataset**

**I. Instruments**

**Instruments.** The F-4500 Fluorescent spectrophotometer (Hitachi Limited)., Mettler FiveEasy28 pH Meter (METTLER TOLEDO), NanoDrop™ One Microvolume UV-Vis Spectrophotometer (ThermoFisher Scientific), DK-8D Three-Hole Electric Thermostatic Water Bath (Yiheng Technology Co., Ltd., Shanghai, China), Electronic balance (Sartorius, Germany), RiOs^TM^8 Water Purification System (Merck, the United States), IKA MS 3 basic (France), PIPETMAN pipette (Gilson, Germany), Eppendorf 5417R ultracentrifuge (Eppendorf, Germany).

**Table S1** In this study, 114 sets of oligonucleotide strands were employed, each comprising strands F, Q, and I. For instance, the initial experiments involved Q-RD (16)/F-RD (16)-1/I-RD (16)-1. The first 98 sequences represent the training and validation sets, while the rest sequences denote the test set.

| **Label** | **Sequence** | **Label** | **Sequence** |
| --- | --- | --- | --- |
| Q-RD (16) | GACGACTAGTACTCGT |  |  |
| F-RD (16)-1 | ACGAGTACTAGTCGTCCTCGGA | I-RD (16)-1 | TCCGAGGACGACTAGTACTCGT |
| F-RD (16)-2 | ACGAGTACTAGTCGTCAGGCGA | I-RD (16)-2 | TCGCCTGACGACTAGTACTCGT |
| F-RD (16)-3 | ACGAGTACTAGTCGTCGAGGTG | I-RD (16)-3 | CACCTCGACGACTAGTACTCGT |
| F-RD (16)-4 | ACGAGTACTAGTCGTCTTGATG | I-RD (16)-4 | CATCAAGACGACTAGTACTCGT |
| F-RD (16)-5 | ACGAGTACTAGTCGTCTGGTTT | I-RD (16)-5 | AAACCAGACGACTAGTACTCGT |
| F-RD (16)-6 | ACGAGTACTAGTCGTCCCGAGC | I-RD (16)-6 | GCTCGGGACGACTAGTACTCGT |
| F-RD (16)-7 | ACGAGTACTAGTCGTCTTAAAC | I-RD (16)-7 | GTTTAAGACGACTAGTACTCGT |
| F-RD (16)-8 | ACGAGTACTAGTCGTCCTCCCG | I-RD (16)-8 | CGGGAGGACGACTAGTACTCGT |
| F-RD (16)-9 | ACGAGTACTAGTCGTCTACCGC | I-RD (16)-9 | GCGGTAGACGACTAGTACTCGT |
| F-RD (16)-10 | ACGAGTACTAGTCGTCAGATCA | I-RD (16)-10 | TGATCTGACGACTAGTACTCGT |
| F-RD (16)-11 | ACGAGTACTAGTCGTCTACCTC | I-RD (16)-11 | GAGGTAGACGACTAGTACTCGT |
| F-RD (16)-12 | ACGAGTACTAGTCGTCGGTACC | I-RD (16)-12 | GGTACCGACGACTAGTACTCGT |
| F-RD (16)-13 | ACGAGTACTAGTCGTCCGCCTC | I-RD (16)-13 | GAGGCGGACGACTAGTACTCGT |
| F-RD (16)-14 | ACGAGTACTAGTCGTCTAGTAG | I-RD (16)-14 | CTACTAGACGACTAGTACTCGT |
| F-RD (16)-15 | ACGAGTACTAGTCGTCAATACC | I-RD (16)-15 | GGTATTGACGACTAGTACTCGT |
| F-RD (16)-16 | ACGAGTACTAGTCGTCGGGTCA | I-RD (16)-16 | TGACCCGACGACTAGTACTCGT |
| F-RD (16)-17 | ACGAGTACTAGTCGTCTAGTCC | I-RD (16)-17 | GGACTAGACGACTAGTACTCGT |
| F-RD (16)-18 | ACGAGTACTAGTCGTCGCCTGT | I-RD (16)-18 | ACAGGCGACGACTAGTACTCGT |
| F-RD (16)-19 | ACGAGTACTAGTCGTCCCGGCG | I-RD (16)-19 | CGCCGGGACGACTAGTACTCGT |
| F-RD (16)-20 | ACGAGTACTAGTCGTCGTAGAG | I-RD (16)-20 | CTCTACGACGACTAGTACTCGT |
| F-RD (16)-21 | ACGAGTACTAGTCGTCACGCAA | I-RD (16)-21 | TTGCGTGACGACTAGTACTCGT |
| F-RD (16)-22 | ACGAGTACTAGTCGTCCCGGAT | I-RD (16)-22 | ATCCGGGACGACTAGTACTCGT |
| F-RD (16)-23 | ACGAGTACTAGTCGTCTCGACC | I-RD (16)-23 | GGTCGAGACGACTAGTACTCGT |
| F-RD (16)-24 | ACGAGTACTAGTCGTCTGTGAG | I-RD (16)-24 | CTCACAGACGACTAGTACTCGT |
| F-RD (16)-25 | ACGAGTACTAGTCGTCCGGATG | I-RD (16)-25 | CATCCGGACGACTAGTACTCGT |
| F-RD (16)-26 | ACGAGTACTAGTCGTCGTTCGA | I-RD (16)-26 | TCGAACGACGACTAGTACTCGT |
| F-RD (16)-27 | ACGAGTACTAGTCGTCAAAGAG | I-RD (16)-27 | CTCTTTGACGACTAGTACTCGT |
| F-RD (16)-28 | ACGAGTACTAGTCGTCTGCACT | I-RD (16)-28 | AGTGCAGACGACTAGTACTCGT |
| F-RD (16)-29 | ACGAGTACTAGTCGTCCCCACT | I-RD (16)-29 | AGTGGGGACGACTAGTACTCGT |
| F-RD (16)-30 | ACGAGTACTAGTCGTCAGGGGA | I-RD (16)-30 | TCCCCTGACGACTAGTACTCGT |
| F-RD (16)-31 | ACGAGTACTAGTCGTCCCCAAG | I-RD (16)-31 | CTTGGGGACGACTAGTACTCGT |
| F-RD (16)-32 | ACGAGTACTAGTCGTCAATACA | I-RD (16)-32 | TGTATTGACGACTAGTACTCGT |
| F-RD (16)-33 | ACGAGTACTAGTCGTCGATCCG | I-RD (16)-33 | CGGATCGACGACTAGTACTCGT |
| F-RD (16)-34 | ACGAGTACTAGTCGTCCGCCCG | I-RD (16)-34 | CGGGCGGACGACTAGTACTCGT |
| F-RD (16)-35 | ACGAGTACTAGTCGTCCCTGAC | I-RD (16)-35 | GTCAGGGACGACTAGTACTCGT |
| F-RD (16)-36 | ACGAGTACTAGTCGTCGGTTCC | I-RD (16)-36 | GGAACCGACGACTAGTACTCGT |
| F-RD (16)-37 | ACGAGTACTAGTCGTCGTCTGG | I-RD (16)-37 | CCAGACGACGACTAGTACTCGT |
| F-RD (16)-38 | ACGAGTACTAGTCGTCCAACGC | I-RD (16)-38 | GCGTTGGACGACTAGTACTCGT |
| F-RD (16)-39 | ACGAGTACTAGTCGTCCTCAGC | I-RD (16)-39 | GCTGAGGACGACTAGTACTCGT |
| F-RD (16)-40 | ACGAGTACTAGTCGTCAATTAC | I-RD (16)-40 | GTAATTGACGACTAGTACTCGT |
| F-RD (16)-41 | ACGAGTACTAGTCGTCTCTGGC | I-RD (16)-41 | GCCAGAGACGACTAGTACTCGT |
| F-RD (16)-42 | ACGAGTACTAGTCGTCAAGGTC | I-RD (16)-42 | GACCTTGACGACTAGTACTCGT |
| F-RD (16)-43 | ACGAGTACTAGTCGTCATATAG | I-RD (16)-43 | CTATATGACGACTAGTACTCGT |
| F-RD (16)-44 | ACGAGTACTAGTCGTCGAGAGA | I-RD (16)-44 | TCTCTCGACGACTAGTACTCGT |
| F-RD (16)-45 | ACGAGTACTAGTCGTCGACCAG | I-RD (16)-45 | CTGGTCGACGACTAGTACTCGT |
| F-RD (16)-46 | ACGAGTACTAGTCGTCGCATTG | I-RD (16)-46 | CAATGCGACGACTAGTACTCGT |
| F-RD (16)-47 | ACGAGTACTAGTCGTCCTGACA | I-RD (16)-47 | TGTCAGGACGACTAGTACTCGT |
| F-RD (16)-48 | ACGAGTACTAGTCGTCCCATTC | I-RD (16)-48 | GAATGGGACGACTAGTACTCGT |
| F-RD (16)-49 | ACGAGTACTAGTCGTCCATCGG | I-RD (16)-49 | CCGATGGACGACTAGTACTCGT |
| F-RD (16)-50 | ACGAGTACTAGTCGTCTCCAAA | I-RD (16)-50 | TTTGGAGACGACTAGTACTCGT |
| F-RD (16)-51 | ACGAGTACTAGTCGTCTGAAAT | I-RD (16)-51 | ATTTCAGACGACTAGTACTCGT |
| F-RD (16)-52 | ACGAGTACTAGTCGTCTCATCC | I-RD (16)-52 | GGATGAGACGACTAGTACTCGT |
| F-RD (16)-53 | ACGAGTACTAGTCGTCCTGGAT | I-RD (16)-53 | ATCCAGGACGACTAGTACTCGT |
| F-RD (16)-54 | ACGAGTACTAGTCGTCTCGCTT | I-RD (16)-54 | AAGCGAGACGACTAGTACTCGT |
| F-RD (16)-55 | ACGAGTACTAGTCGTCATATCG | I-RD (16)-55 | CGATATGACGACTAGTACTCGT |
| F-RD (16)-56 | ACGAGTACTAGTCGTCTCGCAT | I-RD (16)-56 | ATGCGAGACGACTAGTACTCGT |
| F-RD (16)-57 | ACGAGTACTAGTCGTCGGACAG | I-RD (16)-57 | CTGTCCGACGACTAGTACTCGT |
| F-RD (16)-58 | ACGAGTACTAGTCGTCCTACGT | I-RD (16)-58 | ACGTAGGACGACTAGTACTCGT |
| F-RD (16)-59 | ACGAGTACTAGTCGTCGCTGAT | I-RD (16)-59 | ATCAGCGACGACTAGTACTCGT |
| F-RD (16)-60 | ACGAGTACTAGTCGTCGCCCTA | I-RD (16)-60 | TAGGGCGACGACTAGTACTCGT |
| F-RD (16)-61 | ACGAGTACTAGTCGTCGTGACC | I-RD (16)-61 | GGTCACGACGACTAGTACTCGT |
| F-RD (16)-62 | ACGAGTACTAGTCGTCAAACCG | I-RD (16)-62 | CGGTTTGACGACTAGTACTCGT |
| F-RD (16)-63 | ACGAGTACTAGTCGTCCATGGC | I-RD (16)-63 | GCCATGGACGACTAGTACTCGT |
| F-RD (16)-64 | ACGAGTACTAGTCGTCACAGGC | I-RD (16)-64 | GCCTGTGACGACTAGTACTCGT |
| F-RD (16)-65 | ACGAGTACTAGTCGTCTCGCTT | I-RD (16)-65 | AAGCGAGACGACTAGTACTCGT |
| F-RD (16)-66 | ACGAGTACTAGTCGTCGACCAC | I-RD (16)-66 | GTGGTCGACGACTAGTACTCGT |
| F-RD (16)-67 | ACGAGTACTAGTCGTCCCGCAG | I-RD (16)-67 | CTGCGGGACGACTAGTACTCGT |
| F-RD (16)-68 | ACGAGTACTAGTCGTCTCGCGC | I-RD (16)-68 | GCGCGAGACGACTAGTACTCGT |
| F-RD (16)-69 | ACGAGTACTAGTCGTCGAAAAC | I-RD (16)-69 | GTTTTCGACGACTAGTACTCGT |
| F-RD (16)-70 | ACGAGTACTAGTCGTCCTCTGA | I-RD (16)-70 | TCAGAGGACGACTAGTACTCGT |
| F-RD (16)-71 | ACGAGTACTAGTCGTCTTGCGC | I-RD (16)-71 | GCGCAAGACGACTAGTACTCGT |
| F-RD (16)-72 | ACGAGTACTAGTCGTCCTTTTT | I-RD (16)-72 | AAAAAGGACGACTAGTACTCGT |
| F-RD (16)-73 | ACGAGTACTAGTCGTCCGCAGC | I-RD (16)-73 | GCTGCGGACGACTAGTACTCGT |
| F-RD (16)-74 | ACGAGTACTAGTCGTCGCGGTG | I-RD (16)-74 | CACCGCGACGACTAGTACTCGT |
| F-RD (16)-75 | ACGAGTACTAGTCGTCTCCCCC | I-RD (16)-75 | GGGGGAGACGACTAGTACTCGT |
| F-RD (16)-76 | ACGAGTACTAGTCGTCCATTCG | I-RD (16)-76 | CGAATGGACGACTAGTACTCGT |
| F-RD (16)-77 | ACGAGTACTAGTCGTCTGAGTT | I-RD (16)-77 | AACTCAGACGACTAGTACTCGT |
| F-RD (16)-78 | ACGAGTACTAGTCGTCGCCACC | I-RD (16)-78 | GGTGGCGACGACTAGTACTCGT |
| F-RD (16)-79 | ACGAGTACTAGTCGTCAAAAAA | I-RD (16)-79 | TTTTTTGACGACTAGTACTCGT |
| F-RD (16)-80 | ACGAGTACTAGTCGTCTTTCCT | I-RD (16)-80 | AGGAAAGACGACTAGTACTCGT |
| F-RD (16)-81 | ACGAGTACTAGTCGTCACCATT | I-RD (16)-81 | AATGGTGACGACTAGTACTCGT |
| F-RD (16)-82 | ACGAGTACTAGTCGTCCGGAGT | I-RD (16)-82 | ACTCCGGACGACTAGTACTCGT |
| F-RD (16)-83 | ACGAGTACTAGTCGTCGGAGGG | I-RD (16)-83 | CCCTCCGACGACTAGTACTCGT |
| F-RD (16)-84 | ACGAGTACTAGTCGTCGACCAA | I-RD (16)-84 | TTGGTCGACGACTAGTACTCGT |
| F-RD (16)-85 | ACGAGTACTAGTCGTCTGGTGA | I-RD (16)-85 | TCACCAGACGACTAGTACTCGT |
| F-RD (16)-86 | ACGAGTACTAGTCGTCAATTGA | I-RD (16)-86 | TCAATTGACGACTAGTACTCGT |
| F-RD (16)-87 | ACGAGTACTAGTCGTCTACGGA | I-RD (16)-87 | TCCGTAGACGACTAGTACTCGT |
| F-RD (16)-88 | ACGAGTACTAGTCGTCTACTCA | I-RD (16)-88 | TGAGTAGACGACTAGTACTCGT |
| F-RD (16)-89 | ACGAGTACTAGTCGTCTAAAGG | I-RD (16)-89 | CCTTTAGACGACTAGTACTCGT |
| F-RD (16)-90 | ACGAGTACTAGTCGTCGAGTGG | I-RD (16)-90 | CCACTCGACGACTAGTACTCGT |
| F-RD (16)-91 | ACGAGTACTAGTCGTCCACGAA | I-RD (16)-91 | TTCGTGGACGACTAGTACTCGT |
| F-RD (16)-92 | ACGAGTACTAGTCGTCCACGAG | I-RD (16)-92 | CTCGTGGACGACTAGTACTCGT |
| F-RD (16)-93 | ACGAGTACTAGTCGTCCACGGA | I-RD (16)-93 | TCCGTGGACGACTAGTACTCGT |
| F-RD (16)-94 | ACGAGTACTAGTCGTCCACGGG | I-RD (16)-94 | CCCGTGGACGACTAGTACTCGT |
| F-RD (16)-95 | ACGAGTACTAGTCGTCCACAAA | I-RD (16)-95 | TTTGTGGACGACTAGTACTCGT |
| F-RD (16)-96 | ACGAGTACTAGTCGTCCACAAG | I-RD (16)-96 | CTTGTGGACGACTAGTACTCGT |
| F-RD (16)-97 | ACGAGTACTAGTCGTCCACAGA | I-RD (16)-97 | TCTGTGGACGACTAGTACTCGT |
| F-RD (16)-98 | ACGAGTACTAGTCGTCCACAGG | I-RD (16)-98 | CCTGTGGACGACTAGTACTCGT |
| F-RD (16)-99 | ACGTCTACTAGTCGTCCTCGGA | Q-RD (16)-99 | GACGACTAGTAGACGT |
| I-RD (16)-99 | TCCGAGGACGACTAGTAGACGT | F-RD (16)-100 | TCGACTACTAGTCGTCCTCGGA |
| Q-RD (16)-100 | GACGACTAGTAGTCGA | I-RD (16)-100 | TCCGAGGACGACTAGTAGTCGA |
| F-RD (16)-101 | AGGAGTACTAGTCGTCCTCGGA | Q-RD (16)-101 | GACGACTAGTACTCCT |
| I-RD (16)-101 | TCCGAGGACGACTAGTACTCCT | F-RD (16)-102 | AGCAGTACTAGTCGTCCTCGGA |
| Q-RD (16)-102 | GACGACTAGTACTGCT | I-RD (16)-102 | TCCGAGGACGACTAGTACTGCT |
| F-RD (16)-103 | ACGACTACTAGTCGTCCTCGGA | Q-RD (16)-103 | GACGACTAGTAGTCGT |
| I-RD (16)-103 | TCCGAGGACGACTAGTAGTCGT | F-RD (16)-104 | ATGAGTACTAGTCGTCCTCTGA |
| Q-RD (16)-104 | GACGACTAGTACTCAT | I-RD (16)-104 | TCAGAGGACGACTAGTACTCAT |
| F-RD (16)-105 | ATGAGTCCTAGTCGTCCTCTGA | Q-RD (16)-105 | GACGACTAGGACTCAT |
| I-RD (16)-105 | TCAGAGGACGACTAGGACTCAT | F-RD (16)-106 | ATGAGTCTTAGTCGTCCTCTGA |
| Q-RD (16)-106 | GACGACTAAGACTCAT | I-RD (16)-106 | TCAGAGGACGACTAAGACTCAT |
| F-RD (16)-107 | AAGAGTCTTAGTCGTCCTCTGA | Q-RD (16)-107 | GACGACTAAGACTCTT |
| I-RD (16)-107 | TCAGAGGACGACTAAGACTCTT | F-RD (16)-108 | CAGATTCTTAGTCGTCCTCTGA |
| Q-RD (16)-108 | GACGACTAAGAATCTG | I-RD (16)-108 | TCAGAGGACGACTAAGAATCTG |
| F-RD (16)-109 | ACGAGTCTAGTCGTCCTCGGA | Q-RD (16)-109 | GACGACTAGACTCGT |
| I-RD (16)-109 | TCCGAGGACGACTAGACTCGT | F-RD (16)-110 | ACGAGTTAGTCGTCCTCGGA |
| Q-RD (16)-110 | GACGACTAACTCGT | I-RD (16)-110 | TCCGAGGACGACTAACTCGT |
| F-RD (16)-111 | ACGATGTACTAGTCGTCCTCGGA | Q-RD (16)-111 | GACGACTAGTACATCGT |
| I-RD (16)-111 | TCCGAGGACGACTAGTACATCGT | F-RD (16)-112 | ACAGTACTAGTCGTCCTCTGA |
| Q-RD (16)-112 | GACGACTAGTACTGT | I-RD (16)-112 | TCAGAGGACGACTAGTACTGT |
| F-RD (16)-113 | ACAGTACAGTCGTCCTCTGA | Q-RD (16)-113 | GACGACTGTACTGT |
| I-RD (16)-113 | TCAGAGGACGACTGTACTGT | F-RD (16)-114 | ACGAGTATCTAGTCGTCCTCTGA |
| Q-RD (16)-114 | GACGACTAGATACTCGT | F-RD (16)-114 | TCAGAGGACGACTAGATACTCGT |

**II. Experimental Data and Model Fitting**

**Data processing.** A total of 114 experiments were conducted, and the data processing for each group was consistent. The process involved creating a standard curve by plotting the fluorescence intensity measured at different concentrations of the strand IF. This was used to establish a linear relationship between concentration and fluorescence intensity. Next, the dead time was added to the time scale, and the fluorescence intensity of the background was subtracted uniformly from the original kinetic data. Finally, the fluorescence intensity was converted into concentration using the standard curve to obtain the relationship between the change in time and concentration.

**Kinetic fitting.** The TMSD in this study involved a kinetic process of three-branch migration and was modelled using a second-order kinetic equation^1^. The mathematical derivation is as follows.

The reaction formula is

The rate equation of the reaction is

Because strand QF is mixed with strand I in equal volume and concentration:

In this case, the rate equation can be written as:

To separate variables from the above formula, and do the antiderivative, there is:

It follows that

Based on the above formula, when t = 0, [I]_0_ is the initial concentration of strand I, then:

Part of the strand I is bound to the strand QF, but the strand Q is not completely dissociated, and the quenching group is not completely removed, therefore the fluorescence will not be 100% restored. A correction coefficient α is required, and the true concentration of strand I is:

Because the change of fluorescence intensity is manifested as the change of [IF], and:

So, we can get:

Generally, employing lower concentrations of TMSD tends to minimize side reactions throughout the system. Additionally, the reaction concentration should be maintained within the linear range of the fluorescence standard curve for accurate measurements. In this study, a reaction concentration of 50 nM was selected and utilized. By substituting [I]_0_ = 50 nM into the above equation, the following results were obtained:

The software origin 2019b was used to fit the data, using the following equation:

$$\left[ IF \right]=50-\left( \alpha*\frac{1}{kt+\frac{1}{50}}+C \right)$$

In the above equations, [] represents the concentration, for example, [IF] represents the concentration of the IF strand, α is the correction factor, *k* is the rate constant, and C is a constant.


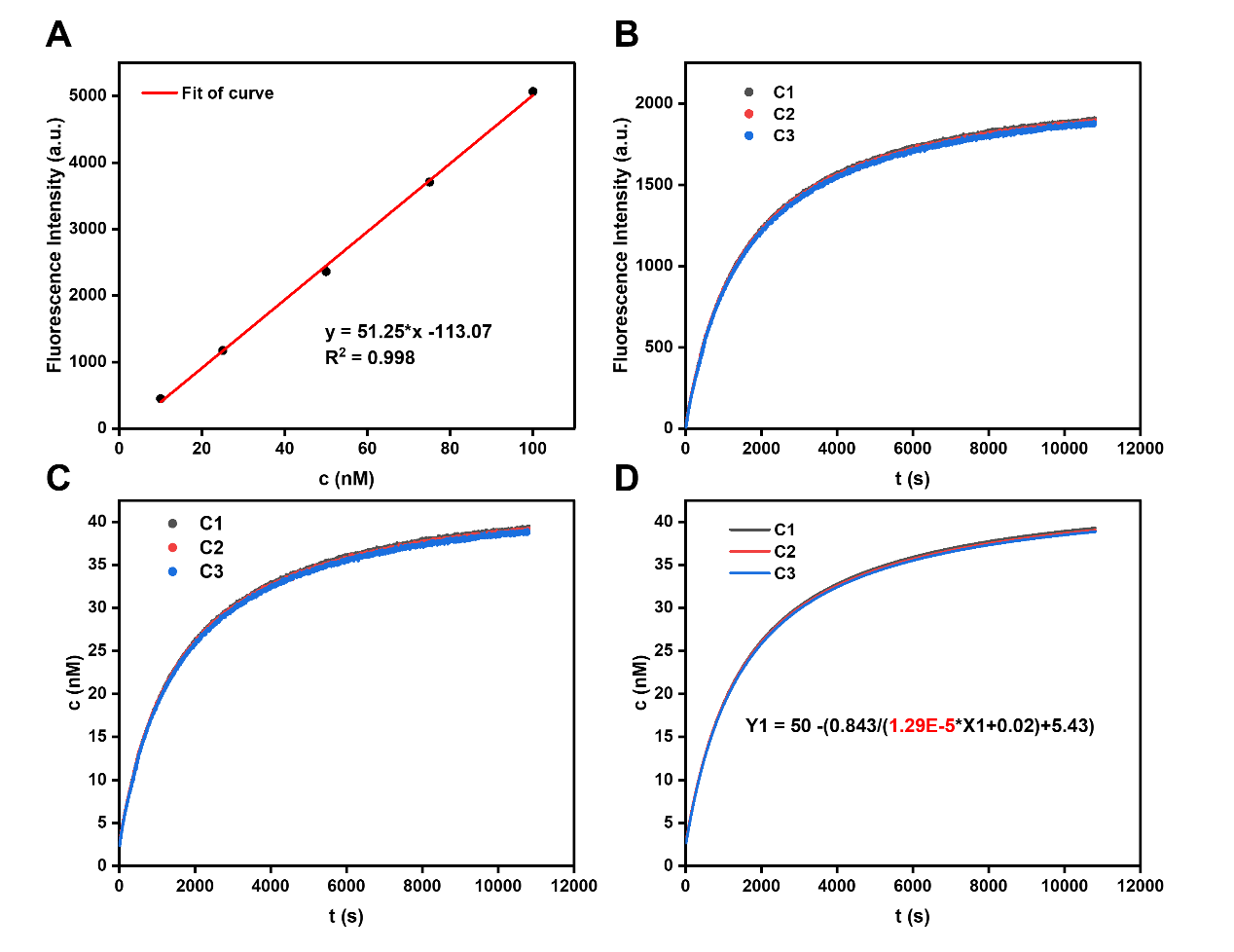


**Figure S1** Data processing process. (**A**) The standard curve of samples; (**B**) Fluorescence kinetic curve; (**C**) Kinetic plots of DNA concentration versus time; (**D**) The fitted kinetic curve.

The results of fitting 114 sets of experimental data are presented below, where C1, C2, and C3 denote three sets of parallel experiments. Because of the high reproducibility, not all experiments were conducted in parallel.

|  |  |  |
| --- | --- | --- |
|  |  |  |
|  |  |  |
|  |  |  |
|  |  |  |
|  |  |  |
|  |  |  |
|  |  |  |
|  |  |  |
|  |  |  |
|  |  |  |
|  |  |  |
|  |  |  |
|  |  |  |
|  |  |  |
|  |  |  |
|  |  |  |
|  |  |  |
|  |  |  |
|  |  |  |
|  |  |  |
|  |  |  |
|  |  |  |
|  |  |  |
|  |  |  |
|  |  |  |
|  |  |  |
|  |  |  |
|  |  |  |
|  |  |  |
|  |  |  |
|  |  |  |
|  |  |  |
|  |  |  |
|  |  |  |
|  |  |  |
|  |  |  |
|  |  |  |

**Figure S2** 114 sets of fitted kinetic curves.

**III. Feature Extraction and Feature List**

**Feature Extraction.** The web page NUPACK version 3 ([www.nupack.org](http://www.nupack.org/))^2^ was used to obtain essential data on the TMSD. The features are mainly about the invader strand and the toehold just-binding ternary intermediates and the substrate strand (Fig. S3). It needs to state that the ternary intermediate is supposed to form by perfect complexation of the toehold domains from the substrate strand and the invader strand.


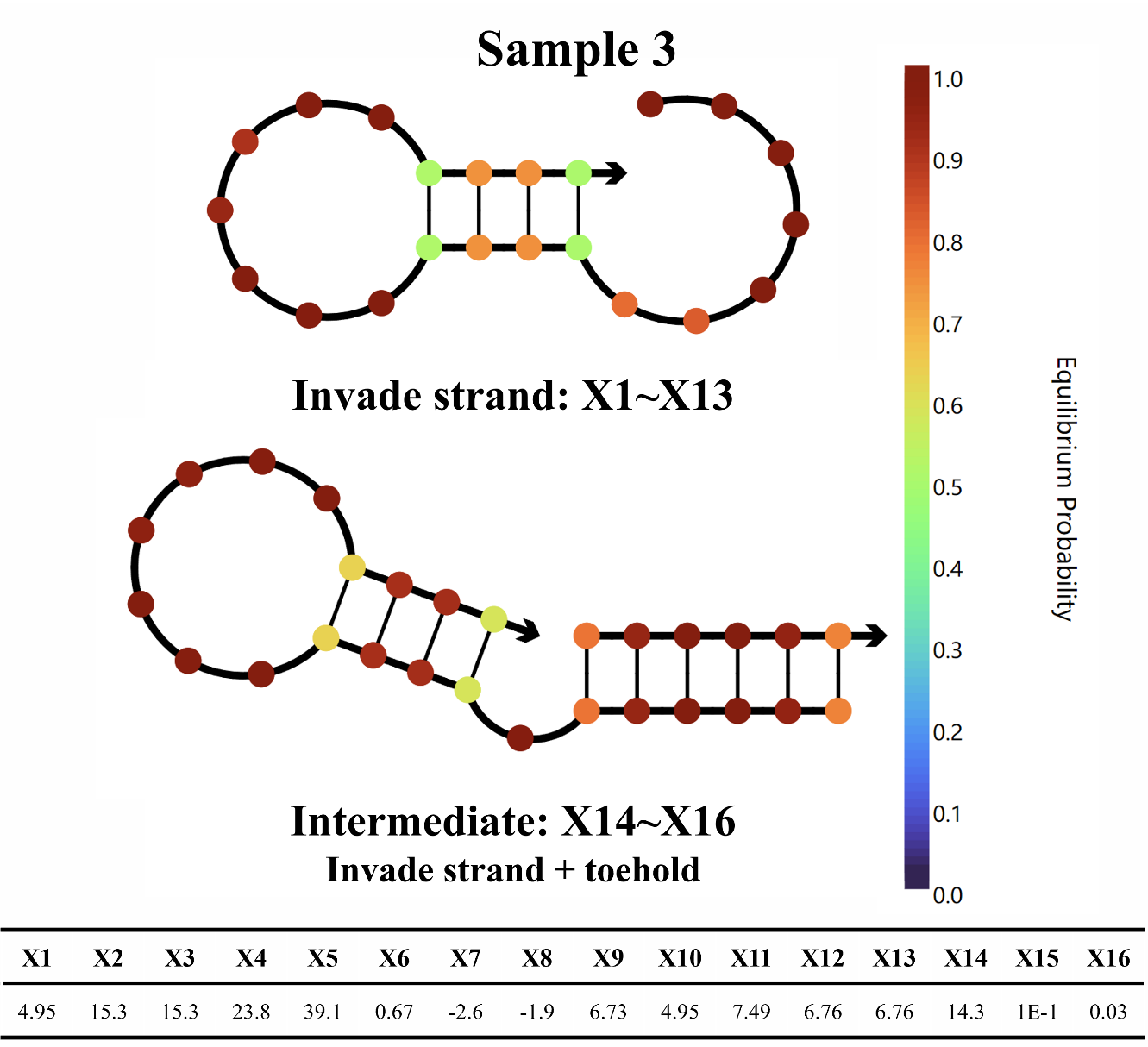


**Figure S3** The source of features X1~X16(Take sample 3 as an example): the schematic diagram of the strand is from NUPACK.

**Variables List.** This involves variable names, descriptions, and ranges of values. All variables involve classic Watson-Crick base pairing for hydrogen bonds, without considering other atypical hydrogen bonds.

**Table S2** The name of each variable, the description of each variable, and the corresponding range of values.

|  | **Variable** | **Variable name** | **Description** | **Range** |
| --- | --- | --- | --- | --- |
| **Structure of the invader strand** | X1 | Number of unpaired bases in toehold in MFE | For the minimum free energy structure of the invading strand, the sum of the probabilities of unpaired bases in the toehold region. | [0.831,5.997] |
|  | X2 | Total free sites in toehold | The unpairing probability of the bases in toehold multiplied by the binding sites of the hydrogen bond (three sites at G/C and two sites at A/T) | [5.081,14.447] |
|  | X3 | The overall free sites in toehold in MFE | The probability of unpaired bases in the MFE structure multiplied by the number of hydrogen bonding sites | [1.662,16.383] |
|  | X4 | free sites of the branch migration region | The unpairing probability of the overall free sites in the branch migration region | [19.303,28.759] |
|  | X5 | overall free sites | The sum of the number of free hydrogen bond sites in the whole strand | [24.384,41.717] |
|  | X6 | The G/C ratio in the toehold region | Take the number of G/C divided by the number of bases in the toehold | [0,1] |
|  | X7 | The free energy of the whole strand I | The NUPACK prediction is used to obtain the free energy of the whole strand | [-5.97, -2.48] |
|  | X8 | The free energy of MFE | The free energy of the minimum free energy structure | [-5.94, -1.97] |
|  | X9 | The sum of pairing probability of all the bases in the branch migration region | The total number of bases minus the total probability of each base being unpaired | [4.936,8.882] |
|  | X10 | The number of bases remaining in the toehold of the strand I | The sum of the unpaired probabilities of all bases in the toehold region of strand I | [1.925,5.997] |
|  | X11 | The number of hydrogen bonds in MFE | The pairing probability of each base in the minimum free energy structure multiplied by the number of hydrogen bonding sites (mathematical expectation) | [2.599,15.793] |
|  | X12 | Number of free bases in the ring | Number of unpaired bases in all hairpin rings (probability) | [2.469,8.745] |
|  | X13 | Number of free bases in the biggest ring | The sum of the unpaired probabilities that all bases in the largest hairpin ring | [2.469,8.745] |
| **Intermediate** | X14 | Bonding of intermediates (number of hydrogen bonds) | The sum of pairing probability of each base that multiplied by corresponding hydrogen bonding number | (0,17.728] |
|  | X15 | The amount of intermediates formed by perfect complexation of the toehold domains from the substrate strand and the invader strand. | The amount of intermediates (ternary complexes) predicted in NUPACK | (0,10.4] |
|  | X16 | Other intermediates formed by base pairing between the branch migration region of the invader strand and the toehold of the QF strand | If the value is less than 1E-5, the default value is none | [0,0.4298] |

**IV. Temperature Dependent Kinetics Experiments**

**Determined Energy Barriers of the TMSD.** Temperature-variable kinetics experiments were conducted to test the activation energies of three samples. For each sample, a temperature gradient of 15°C, 20°C, 25°C, and 30°C was selected (Fig. S4A-4C). The rate constants were obtained by fitting to a second-order kinetic equation (part II). The activation energies were derived by fitting to the Arrhenius equation (Fig. S4D-4F).


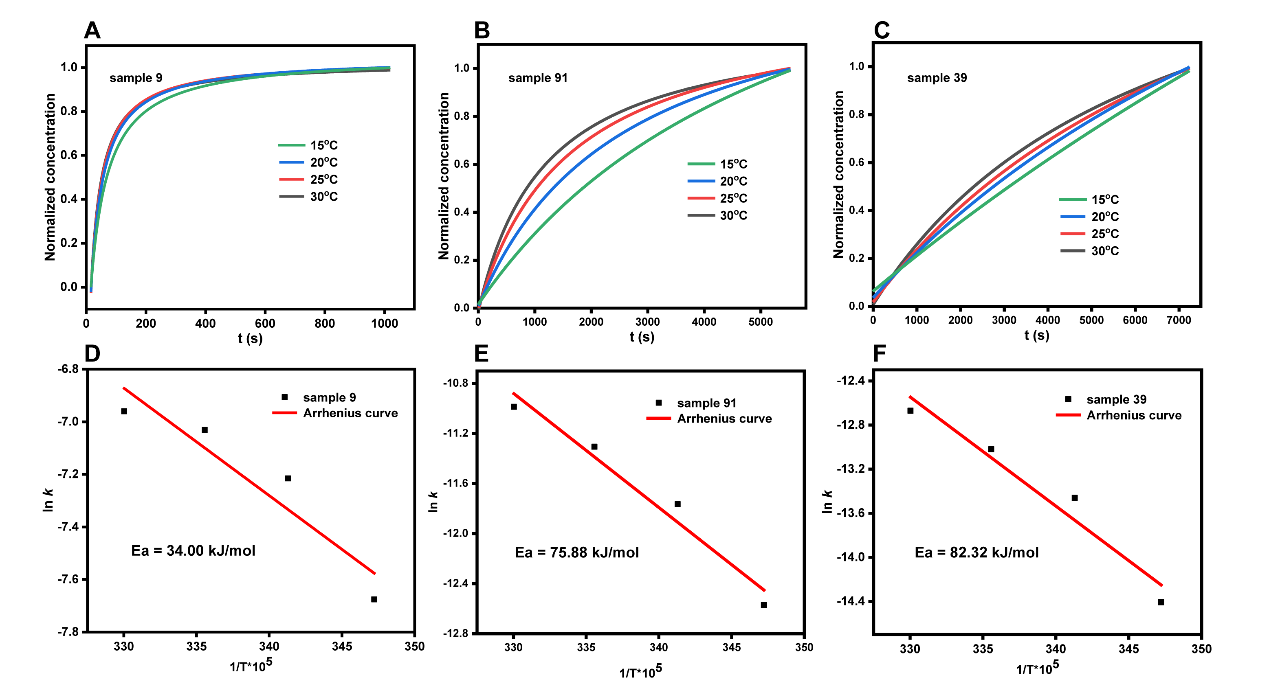


**Figure S4** Experimental data of temperature-variable kinetics. (**A**, **B**, **C**) The displacement kinetics curves of samples **9**, **91**, **39** at 15℃, 20℃, 25℃, 30℃, respectively. (**D**, **E**, **F**) The Arrhenius curves and the calculated activation energies of samples **9**, **91**, **39**.

**Calculation of** **Gibbs free energy.** NUPACK is used to calculate the Gibbs free energy. After setting the model option according to the experimental conditions, strand I and strand Q were input to calculate the Gibbs free energy of the two single strands. I strand and F strand, Q strand and F strand were input to calculate the free energy of IF strand and QF strand, and the free energy change of the strand displacement reaction was obtained. The results are listed in Table S3.

**Table S3** The free energy of each sample, the free energy change of the total strand displacement reaction and the activation energies.

| **Sample** | **I**  **(Kcal/mol)** | **QF**  **(Kcal/mol)** | **IF**  **(Kcal/mol)** | **Q**  **(Kcal/mol)** | **ΔG**  **(Kcal/mol)** | $\boldsymbol{E}_{\boldsymbol{a}}$  **(KJ/mol)** |
| --- | --- | --- | --- | --- | --- | --- |
| Sample 9 | -2.92 | -24.59 | -34.5 | -2.48 | -9.47 | 34.00 |
| Sample 91 | -3.31 | -24.32 | -34.18 | -2.48 | -9.03 | 75.88 |
| Sample 39 | -3.56 | -24.32 | -34.28 | -2.48 | -8.88 | 82.32 |

**V. Machine Learning and Database**

**Code Environment.** The machine learning part was mainly implemented in Python 3.9.12 in jupyter notebook 6.4.12. The data analysis used some Python packages such as numpy 1.23.4, scikit-learn 1.0.2, pandas 1.5.1, matplotlib 3.6.2.

**Machine Learning Theory.** The paper primarily employed three machine learning algorithms: decision tree, support vector machine, and logistic regression.

The decision tree algorithm is renowned for its robust interpretability and rapid computation speed. The main drawbacks of the decision tree algorithm are its susceptibility to overfitting, sensitivity to noise, and poor stability^3^. Typically, it operates by sorting data through a process akin to answering a sequence of questions. The splitting algorithm's objective function is designed to maximize information gain at each bifurcation. The concept of information gain is defined as follows:

Where, *f* is the feature used to split the data, represents the parent node, represents the *j ^th^* child node, *I* is the impurity content, is the sample number of the parent node, and is the sample number of the *j ^th^* child node. In binary decision trees, entropy (*I_H_*), Gini impurity (*I_G_*), and classification error (*I_E_*) are the three commonly used metrics to measure impurity content. The definition of using entropy as a measure is as follows:

In which $p\left( \left. i \right|t \right)\neq0$represents the probability of a certain node t belonging to the i class of samples. The primary objective of using the Gini impurity is to minimize the probability of misclassification, and it is defined as follows:

Another method is the classification error method, which is defined as:

The Gini impurity was used as the splitting criterion for the decision tree in this paper.

Logistic regression is a widely used method in statistical analysis that provides a quantification of the impact of variables through intuitive odds ratios, suitable for binary classification problems, robust to outliers, computationally efficient, and easy to implement; however, it is also limited by its assumption of linear relationships between variables, susceptible to multicollinearity, not flexible enough for complex nonlinear relationships, at risk of overfitting, and its accuracy relies on the quality and completeness of the input data^4^. For multi-class classification problems, the algorithm can be adapted by using a modified function. Therefore, the training set is assumed to be, where . In general, given the feature value x, the logistic regression algorithm computes the probability that a sample belongs to each of the categories, and the sum of the probabilities of all the categories is equal to 1. The principle is as follows:

among them, are the parameter in the model, is a normalized term.

Support Vector Machine (SVM) offers excellent classification and predictive capabilities, particularly with the kernel trick that effectively addresses nonlinear issues; however, they fall short compared to generalized linear models in intuitively explaining the relationships between data, necessitating additional post-hoc analysis to thoroughly comprehend the predictive capacity of the variables involved^5^. The support vector machine (SVM) algorithm can be regarded as an extension of perceptron. While the perceptron minimizes classification errors, the SVM maximizes classification margins. The classification margin is primarily the distance between the decision boundary and its nearest sample, and the training sample closest to the hyperplane is called the support vector. For multi-classification problems, the support vector machine mainly adopts the one-to-one approach, also known as pair classification. Suppose that a training set containing N samples is given, , in which *K*-dimension feature vector , class label , . For solving class *i* and class *j* data, it can be regarded as a binary support vector machine, namely solving a quadratic programming problem. The principle is as follows:

Where, the superscript represents the parameter of the interval equation between class *i* and class *j*, the subscript *t* represents the index of class *i* and class *j* samples. In this paper, represents the mapping relationship between input space and feature space, also known as kernel function.

**Dataset construction.** We have built a database of 114 sets of base sequences corresponding to the rate constants. The rate constants for most sequences are concentrated in the range of 0-0.001 L/(s*nmol), with the maximum value of 4.45E-3 L/(s*nmol) and the minimum value of 1.96E-7 L/(s*nmol), which are about four orders of magnitude different. As shown in Figure 1(B), a classification model was sought to be constructed with the aid of machine learning methods to summarize the factors affecting the rate constants using small sample data. All data were classified into three categories according to the magnitude of the rate constants, the first category (class **0**): [5E-4, ∞) the second category (class **1**): (5E-4, 5E-5) the third category (class **2**): (0, 5E-5]. The classification was based on the time scale of TMSD.

DNA sequences are not to be taken directly as input and need to be encoded. Current coding methods include one-hot coding, sequential coding and k-mer counting^6^. The variables constructed by these coding methods do not properly describe the TMSD systems. For chemical systems, the variables need to be related to the target variables and are usually numerical information which is interpretable and chemically meaningful. The open webpage (www.nupack.org) was used to obtain important thermodynamic data on strand displacement as variables, which were used as input to construct a classification model. Table S2 shows how each variable was constructed, and the range of values taken for each variable. Thus, after transforming the sequence information, we ended up with a 114-item, 16-dimensional dataset.

**Model selection.** Machine learning algorithms can be categorized into four types: supervised learning, semi-supervised learning, unsupervised learning, and reinforcement learning. In this paper, supervised machine learning was employed. In this method, samples had labels, and the algorithm mainly used the mapping relationship between features and target variables. When the target variable was continuous, the learning task was called regression. When the target variable was discrete, the learning task was referred to as classification. This study mainly used three machine learning algorithms: decision tree, support vector machine, and logistic regression.

**Decision tree.** The top three features in terms of importance, X2, X5 and X14, were used to take 73 data for training and 25 data for validation. Cross-validation was used to find the optimal hyperparameters, and the optimal accuracy was about 0.84. To avoid unbalanced data distribution, the total sample was three-fold cross-validation and an accuracy of 0.77 (+/-0.17) was obtained. The visualized decision tree also shows that the number of free hydrogen bonding sites in the entire invader strand is used as the first node and then classified with a maximum tree depth of 3.

**Table S4** Classification report of the decision tree algorithm.

|  | **Precision** | **Recall** | **F1-score** | **Support** |
| --- | --- | --- | --- | --- |
| Class 0 | 1.00 | 0.71 | 0.83 | 7 |
| Class 1 | 0.73 | 1.00 | 0.85 | 11 |
| Class 2 | 1.00 | 0.71 | 0.83 | 7 |
| Accuracy |  |  | 0.84 | 25 |
| Macro avg. | 0.91 | 0.81 | 0.84 | 25 |
| Weighted avg. | 0.88 | 0.84 | 0.84 | 25 |


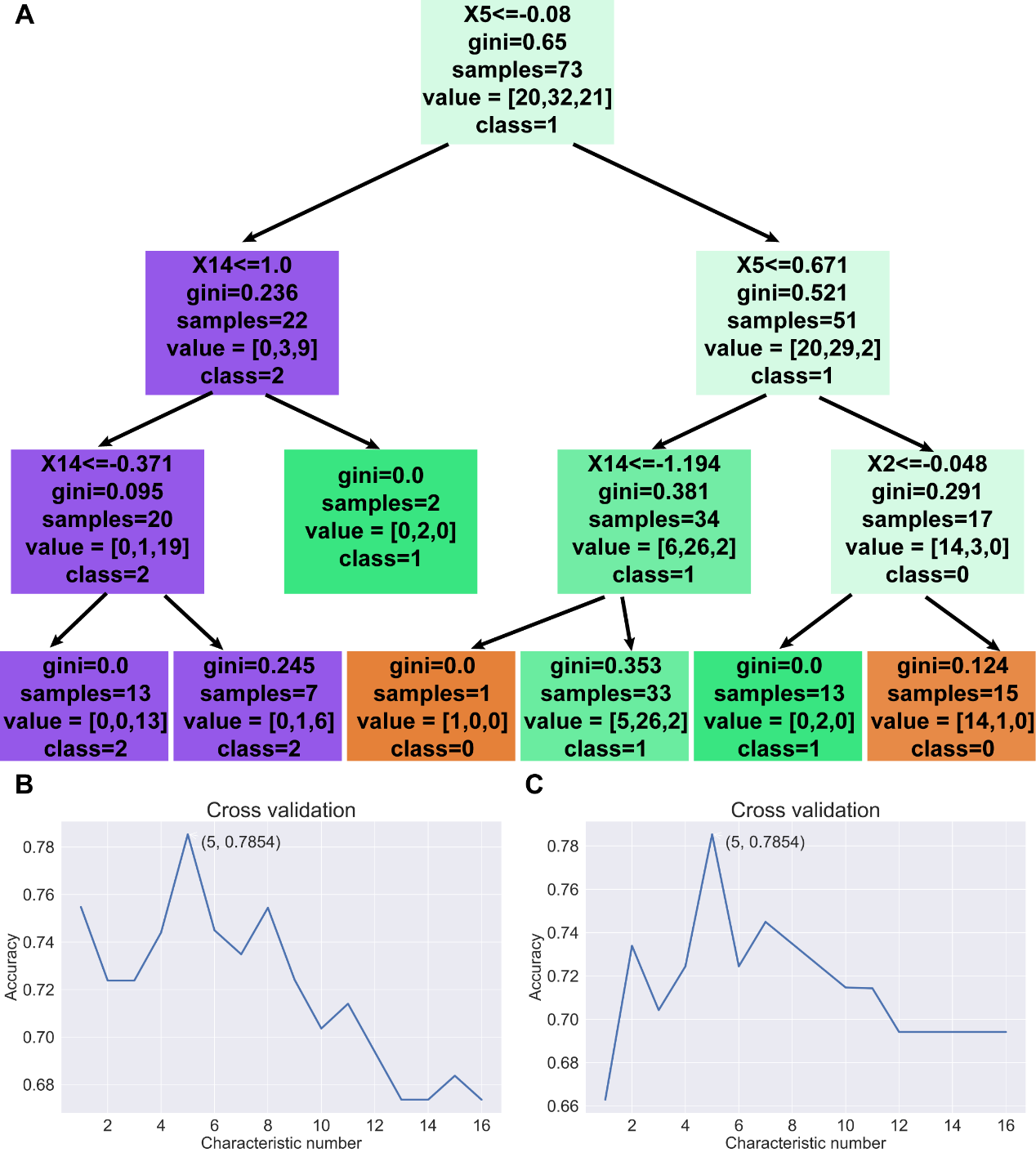


**Figure S5** This figure presents a decision tree visualization alongside analysis of how the accuracy of logistic regression and support vector machine models varies with changes in the number of features. (**A**) Decision tree visualization. (**B**) The relationship between feature number and accuracy of logistic regression. (**C**) The relationship between feature number and accuracy of support vector machine.

**Logistic regression algorithm.** The recursive elimination method was employed to selectively filter out five features with high accuracy, namely X2, X5, X8, X11, and X15. A training set comprising 68 data points and a validation set of 30 data points were utilized. Cross-validation was employed to identify the optimal hyperparameters, resulting in an accuracy of approximately 0.83. Subsequently, three-fold cross-validation was conducted on the entire dataset, yielding an accuracy of 0.79 (±0.14).

**Table S5** Classification report of the logistic regression algorithm.

|  | **Precision** | **Recall** | **F1-score** | **Support** |
| --- | --- | --- | --- | --- |
| Class 0 | 0.78 | 0.88 | 0.82 | 8 |
| Class 1 | 0.83 | 0.77 | 0.80 | 13 |
| Class 2 | 0.89 | 0.89 | 0.89 | 9 |
| Accuracy |  |  | 0.83 | 30 |
| Macro avg | 0.83 | 0.84 | 0.84 | 30 |
| Weighted avg | 0.84 | 0.83 | 0.83 | 30 |

**Support vector machine algorithm.** The recursive elimination method was used to screen six features with high accuracy, which were X2, X5, X6, X8 and X11. The model was trained on a dataset of 68 items and verified on a dataset of 30 items. The optimal hyperparameter was determined by cross-validation and the resulting accuracy was 0.83. Three-fold cross-validation was performed on the full dataset and the accuracy was found to be 0.74 (+/-0.07).

**Model evaluation.** ROC curves are vital metrics for evaluating the effectiveness of classification models. As depicted in Figures 4A, 4B, and 4C, the area under the curves approaches 1 in the upper-left corner, indicating robust classification performance for each category, with AUC values exceeding 0.8. Consequently, these models demonstrate superior categorization abilities. Upon comprehensive evaluation, all three models achieve AUC values surpassing 0.8 on both the training and validation sets, highlighting their high effectiveness in handling small sample sizes.

**Table S6** Comparison of various models.

|  | LR | SVR | DT |
| --- | --- | --- | --- |
| Variable combination | X2/X5/X8/X11/X15 | X2/X5/X6/X8/X11 | X2/X5/X14 |
| Train/Validate accuracy | 0.808/0.833 | 0.838/0.833 | 0.876/0.840 |
| Cross-validation accuracy | 0.79 (+/-0.14) | 0.74 (+/-0.07) | 0.77 (+/-0.17) |

**Table S7** The predictive performance of three algorithms—Decision Tree, Logistic Regression, and Support Vector Machine—is compared against ground truth. Examples of correct predictions are marked in bold for clarity.

| Sample ID | Ground Truth | **Decision tree** | **Logistic regression** | **Support vector machine** |
| --- | --- | --- | --- | --- |
| 99 | 1 | 2 | **1** | **2**2 |
| 100 | 2 | 1 | 1 | **2** |
| 101 | 2 | **2** | **2** | **2** |
| 102 | 1 | **1** | **1** | **1** |
| 103 | 2 | 1 | 1 | **2** |
| 104 | 2 | **2** | **2** | **2** |
| 105 | 2 | **2** | **2** | 1 |
| 106 | 2 | **2** | **2** | 1 |
| 107 | 2 | **2** | **2** | **2** |
| 108 | 2 | **2** | **2** | 1 |
| 109 | 2 | **2** | **2** | **2** |
| 110 | 1 | **1** | **1** | 2 |
| 111 | 2 | 1 | 1 | **2** |
| 112 | 2 | **2** | **2** | **2** |
| 113 | 2 | **2** | **2** | **2** |
| 114 | 2 | 0 | 1 | 0 |
| Accuracy | | **11/16** | **12/16** | **10/16** |

**Machine Learning Model Analysis.** Due to the small sample size and the multi-label classification, logistic regression, support vector machines and decision trees were selected to analyze the data. Both logistic regression and support vector machine algorithms used recursive feature elimination algorithms for feature selection, while the decision tree used the three most important features filtered by random forest. The dataset was then sliced and diced for hyperparameter search, followed by solving for the evaluation parameters such as model accuracy, classification reports, and ROC curves, respectively. Also, to make the model more plausible, additional sequences were designed and the model was tested without changing any parameters. A variety of criteria confirmed that the model is highly accurate and generalizable and can be used as a rate prediction tool for DNA strand displacements.

**Dataset.** All the data are shown in Table S8.

**Table S8** Dataset.

| **Group** | **Sequence (strand I)** | **X1** | **X2** | **X3** | **X4** | **X5** | **X6** | **X7** | **X8** | **X9** | **X10** | **X11** | **X12** | **X13** | **X14** | **X15** | **X16** | **Rate constant L/ (s*nmol）** | **Label (0/1/2)** |
| --- | --- | --- | --- | --- | --- | --- | --- | --- | --- | --- | --- | --- | --- | --- | --- | --- | --- | --- | --- |
| 1 | TCCGAGGACGACTAGTACTCGT | 1.9981 | 5.080619 | 4.9943 | 19.3033 | 24.383919 | 0.66666667 | -5.97 | -5.94 | 8.8816 | 2.02876 | 15.7927 | 6.992 | 4.9928 | 14.07087 | 0.00251 | 0 | 8.08E-07 | 2 |
| 2 | TCGCCTGACGACTAGTACTCGT | 4.1439 | 13.494 | 13.494 | 26.7011 | 40.1951 | 0.6667 | -2.79 | -1.97 | 5.641 | 4.1439 | 6.31765 | 6.7771 | 6.7771 | 13.7844 | 2.9 | 0.018363 | 2.13E-03 | 0 |
| 3 | CACCTCGACGACTAGTACTCGT | 4.9542 | 15.3313 | 15.3313 | 23.8371 | 39.1684 | 0.67 | -2.62 | -1.97 | 6.73263 | 4.9542 | 7.4924 | 6.7633 | 6.7633 | 14.3664 | 0.111 | 0.0291 | 2.31E-03 | 0 |
| 4 | CATCAAGACGACTAGTACTCGT | 4.9595 | 13.8331 | 13.8331 | 23.6156 | 37.4487 | 0.3333 | -2.52 | -1.97 | 6.72575 | 4.9595 | 7.85608 | 6.7877 | 6.7877 | 12.30985 | 0.0154 | 0.00348 | 1.22E-04 | 1 |
| 5 | AAACCAGACGACTAGTACTCGT | 4.9406 | 13.7639 | 13.7639 | 23.6941 | 37.458 | 0.3333 | -2.52 | -1.97 | 6.69936 | 4.9406 | 7.75419 | 6.7361 | 6.7361 | 11.93759 | 0.0755 | 0.00323 | 2.35E-04 | 1 |
| 6 | GCTCGGGACGACTAGTACTCGT | 2.733 | 11.8271 | 7.3901 | 28.6483 | 40.4754 | 0.8333 | -3.3 | -2.61 | 5.0129 | 3.3246 | 4.82385 | 2.5734 | 2.5734 | 15.4951 | 0.0006 | 0.031652 | 3.25E-04 | 1 |
| 7 | GTTTAAGACGACTAGTACTCGT | 4.9653 | 12.8647 | 12.8647 | 23.5877 | 36.4524 | 0.1667 | -2.51 | -1.97 | 6.73712 | 4.9653 | 7.89108 | 6.7846 | 6.7846 | 11.48267 | 0.00246 | 0.00353 | 1.29E-05 | 2 |
| 8 | CGGGAGGACGACTAGTACTCGT | 2.879 | 10.8149 | 8.637 | 22.8793 | 33.6942 | 0.8333 | -3.45 | -3.18 | 7.49992 | 3.4731 | 10.4337 | 6.5898 | 4.602 | 15.3739 | 0.198 | 0 | 1.27E-05 | 2 |
| 9 | GCGGTAGACGACTAGTACTCGT | 0.9942 | 12.6886 | 2.9826 | 27.1849 | 39.8735 | 0.6667 | -2.92 | -2.1 | 5.504 | 3.9246 | 3.7818 | 4.2606 | 4.2606 | 11.9924 | 0.89 | 0.0157 | 9.26E-04 | 0 |
| 10 | TGATCTGACGACTAGTACTCGT | 4.9628 | 13.8755 | 13.8755 | 23.5202 | 37.3957 | 0.3333 | -2.5 | -1.97 | 6.75523 | 4.9628 | 7.98305 | 6.8101 | 6.8101 | 11.7679 | 0.0554 | 0.02023 | 1.61E-04 | 1 |
| 11 | GAGGTAGACGACTAGTACTCGT | 4.6112 | 13.7779 | 13.7779 | 24.4059 | 38.1838 | 0.5 | -2.61 | -1.97 | 6.5235 | 4.6112 | 6.8517 | 6.3757 | 6.3757 | 12.4781 | 0.0692 | 0.00282 | 9.80E-05 | 1 |
| 12 | GGTACCGACGACTAGTACTCGT | 0.9109 | 11.6234 | 2.7327 | 25.3389 | 36.9623 | 0.6667 | -2.97 | -1.98 | 6.3468 | 3.4178 | 3.7548 | 6.6441 | 6.6441 | 14.77674 | 0.0336 | 0.0492 | 2.31E-04 | 1 |
| 13 | GAGGCGGACGACTAGTACTCGT | 4.6309 | 15.5663 | 15.5663 | 24.5639 | 40.1302 | 0.8333 | -2.65 | -1.97 | 6.4442 | 4.6309 | 6.96005 | 6.4419 | 6.4419 | 16.1019 | 1.66 | 0.00331 | 6.20E-04 | 0 |
| 14 | CTACTAGACGACTAGTACTCGT | 1.8514 | 5.25062 | 4.6978 | 25.0749 | 30.32552 | 0.3333 | -4.48 | -4.26 | 6.95589 | 2.06496 | 11.2218 | 3.8854 | 3.8854 | 7.828754 | 0.000515 | 0.00255 | 1.20E-05 | 2 |
| 15 | GGTATTGACGACTAGTACTCGT | 4.7591 | 13.4158 | 13.4158 | 23.8683 | 37.2841 | 0.3333 | -2.53 | -1.97 | 6.66799 | 4.7591 | 7.55029 | 6.6081 | 6.6081 | 12.55735 | 0.01 | 0.00339 | 7.77E-05 | 1 |
| 16 | TGACCCGACGACTAGTACTCGT | 4.9558 | 15.3381 | 15.3381 | 23.8817 | 39.2198 | 0.6667 | -2.62 | -1.97 | 6.71068 | 4.9558 | 7.50922 | 6.7631 | 6.7631 | 14.1369 | 0.429 | 0.0264 | 2.81E-03 | 0 |
| 17 | GGACTAGACGACTAGTACTCGT | 1.6724 | 5.68751 | 5.0172 | 24.4478 | 30.13531 | 0.5 | -4.37 | -3.95 | 7.10314 | 1.92517 | 11.0863 | 3.8664 | 3.8664 | 12.43414 | 0.00343 | 0.00246 | 4.69E-05 | 2 |
| 18 | ACAGGCGACGACTAGTACTCGT | 4.8859 | 14.2284 | 14.2284 | 24.1257 | 38.3541 | 0.6667 | -2.69 | -1.97 | 6.6554 | 4.8859 | 7.19705 | 6.7085 | 6.7085 | 14.4765 | 0.63 | 0.0396 | 6.79E-04 | 0 |
| 19 | CGCCGGGACGACTAGTACTCGT | 2.8958 | 13.572 | 8.6874 | 28.1445 | 41.7165 | 1 | -3.17 | -2.57 | 5.15635 | 3.6686 | 4.48223 | 2.4693 | 2.4693 | 17.3568 | 6.26 | 0.10956 | 9.51E-04 | 0 |
| 20 | CTCTACGACGACTAGTACTCGT | 3.9826 | 11.8218 | 9.9546 | 23.7976 | 35.6194 | 0.5 | -3.19 | -2.28 | 6.63825 | 4.4437 | 7.70628 | 8.4745 | 8.4745 | 12.8678 | 0.00369 | 0.277 | 1.13E-04 | 1 |
| 21 | TTGCGTGACGACTAGTACTCGT | 4.5101 | 13.4955 | 13.4955 | 24.3921 | 37.8876 | 0.5 | -2.66 | -1.97 | 6.4656 | 4.5101 | 7.01065 | 6.5216 | 6.5216 | 13.0897 | 0.968 | 0.005827 | 2.86E-04 | 1 |
| 22 | ATCCGGGACGACTAGTACTCGT | 2.9822 | 11.7623 | 6.9557 | 28.2087 | 39.971 | 0.6667 | -3.44 | -2.57 | 5.13418 | 3.7176 | 4.55277 | 2.482 | 2.482 | 14.2327 | 0.235 | 0.0908 | 2.23E-04 | 1 |
| 23 | GGTCGAGACGACTAGTACTCGT | 2.4919 | 7.70384 | 6.7295 | 22.8911 | 30.59494 | 0.6667 | -4.44 | -3.65 | 7.2581 | 2.61968 | 10.8654 | 5.1209 | 4.1411 | 14.06859 | 0.0321 | 0.00409 | 2.03E-05 | 2 |
| 24 | CTCACAGACGACTAGTACTCGT | 4.8835 | 14.5878 | 14.5878 | 23.712 | 38.2998 | 0.5 | -2.55 | -1.97 | 6.69457 | 4.8835 | 7.71171 | 6.7521 | 6.7521 | 12.9331 | 0.107 | 0.0037 | 2.10E-04 | 1 |
| 25 | CATCCGGACGACTAGTACTCGT | 4.8807 | 15.2585 | 15.2585 | 23.8301 | 39.0886 | 0.6667 | -2.58 | -1.97 | 6.68372 | 4.8807 | 7.56193 | 6.6307 | 6.6307 | 15.117 | 0.249 | 0.0243 | 2.03E-04 | 1 |
| 26 | TCGAACGACGACTAGTACTCGT | 0.9518 | 11.131 | 1.9036 | 25.8997 | 37.0307 | 0.5 | -3.21 | -2 | 5.9944 | 3.983 | 2.64645 | 3.2944 | 3.2944 | 13.3308 | 0.058 | 0.10301 | 1.72E-04 | 1 |
| 27 | CTCTTTGACGACTAGTACTCGT | 4.9546 | 13.8745 | 13.8745 | 23.3963 | 37.2708 | 0.3333 | -2.5 | -1.97 | 6.79703 | 4.9546 | 8.04075 | 6.83 | 6.83 | 12.49107 | 0.0133 | 0.00342 | 9.02E-05 | 1 |
| 28 | AGTGCAGACGACTAGTACTCGT | 4.5205 | 13.6147 | 13.6147 | 24.0516 | 37.6663 | 0.5 | -2.59 | -1.97 | 6.6521 | 4.5205 | 7.01465 | 6.3249 | 6.3249 | 12.6691 | 0.694 | 0.00647 | 3.22E-04 | 1 |
| 29 | AGTGGGGACGACTAGTACTCGT | 3.8463 | 12.8286 | 12.8286 | 25.8126 | 38.6412 | 0.6667 | -2.78 | -1.97 | 6.10324 | 3.8463 | 5.95716 | 6.129 | 6.129 | 14.3202 | 0.358 | 0.00363 | 1.99E-04 | 1 |
| 30 | TCCCCTGACGACTAGTACTCGT | 4.9432 | 15.8228 | 15.8228 | 23.4748 | 39.2976 | 0.6667 | -2.51 | -1.97 | 6.77102 | 4.9432 | 7.98113 | 6.8264 | 6.8264 | 13.5718 | 1.18 | 0.00345 | 1.59E-03 | 0 |
| 31 | CTTGGGGACGACTAGTACTCGT | 4.8442 | 15.2012 | 15.2012 | 23.8897 | 39.0909 | 0.6667 | -2.59 | -1.97 | 6.67206 | 4.8442 | 7.46644 | 6.5732 | 6.5732 | 14.8759 | 0.153 | 0.00362 | 5.66E-05 | 1 |
| 32 | TGTATTGACGACTAGTACTCGT | 4.7275 | 12.3398 | 12.3398 | 23.938 | 36.2778 | 0.1667 | -2.54 | -1.97 | 6.6492 | 4.7275 | 7.4736 | 6.5763 | 6.5763 | 10.83982 | 0.00947 | 0.00341 | 3.16E-05 | 2 |
| 33 | CGGATCGACGACTAGTACTCGT | 4.7301 | 14.468 | 14.468 | 24.0949 | 38.5629 | 0.6667 | -2.7 | -1.97 | 6.6324 | 4.7301 | 7.2983 | 6.7714 | 6.7714 | 15.0624 | 0.113 | 0.0271 | 1.26E-03 | 0 |
| 34 | CGGGCGGACGACTAGTACTCGT | 4.6194 | 16.3386 | 16.3386 | 24.3267 | 40.6653 | 1 | -2.67 | -1.97 | 6.52843 | 4.6194 | 7.12285 | 6.4924 | 6.4924 | 17.7278 | 10.4 | 0.00388 | 5.95E-04 | 0 |
| 35 | GTCAGGGACGACTAGTACTCGT | 4.4627 | 14.3623 | 14.3623 | 24.7288 | 39.0911 | 0.6667 | -2.65 | -1.97 | 6.39205 | 4.4627 | 6.92008 | 6.4931 | 6.4931 | 14.9957 | 0.12 | 0.00496 | 2.03E-04 | 1 |
| 36 | GGAACCGACGACTAGTACTCGT | 4.7069 | 14.6021 | 14.6021 | 23.7644 | 38.3665 | 0.6667 | -2.7 | -1.97 | 6.74278 | 4.7069 | 7.60347 | 6.7708 | 6.7708 | 14.8665 | 0.0995 | 0.0231 | 1.35E-03 | 0 |
| 37 | CCAGACGACGACTAGTACTCGT | 3.9695 | 13.3235 | 10.9129 | 23.8722 | 37.1957 | 0.6667 | -3.05 | -1.97 | 6.65856 | 4.9707 | 7.17214 | 8.3223 | 8.3223 | 14.74664 | 0.047 | 0.212 | 3.38E-04 | 1 |
| 38 | GCGTTGGACGACTAGTACTCGT | 0.9936 | 10.689 | 2.9808 | 27.188 | 37.877 | 0.6667 | -3.24 | -2.27 | 5.58969 | 4.1392 | 5.53254 | 2.762 | 2.762 | 15.1584 | 0.284 | 0.0231 | 8.21E-05 | 1 |
| 39 | GCTGAGGACGACTAGTACTCGT | 2.846 | 9.1502 | 7.5398 | 22.2987 | 31.4489 | 0.6667 | -3.56 | -3.27 | 7.74976 | 3.4462 | 11.0934 | 6.692 | 4.7077 | 15.0711 | 0.0405 | 0 | 2.75E-06 | 2 |
| 40 | GTAATTGACGACTAGTACTCGT | 5.8741 | 12.7123 | 12.7123 | 23.5094 | 36.2217 | 0.1667 | -2.51 | -1.97 | 6.77685 | 5.8741 | 7.85753 | 6.7153 | 6.7153 | 11.52707 | 0.0025 | 0.00371 | 3.48E-05 | 2 |
| 41 | GCCAGAGACGACTAGTACTCGT | 5.5369 | 14.8184 | 14.8184 | 23.6649 | 38.4833 | 0.6667 | -2.65 | -1.97 | 6.7839 | 5.5369 | 7.0826 | 6.4144 | 6.4144 | 14.396 | 0.594 | 0.00289 | 7.34E-05 | 1 |
| 42 | GACCTTGACGACTAGTACTCGT | 5.9094 | 14.77 | 14.77 | 23.5804 | 38.3504 | 0.5 | -2.52 | -1.97 | 6.73302 | 5.9094 | 7.88198 | 6.7827 | 6.7827 | 11.105 | 0.0733 | 0.00331 | 5.36E-04 | 0 |
| 43 | CTATATGACGACTAGTACTCGT | 5.9552 | 12.8903 | 12.8903 | 23.3874 | 36.2777 | 0.1667 | -2.5 | -1.97 | 6.79947 | 5.9552 | 8.05316 | 6.8272 | 6.8272 | 9.625824 | 0.00117 | 0.00378 | 9.08E-06 | 2 |
| 44 | TCTCTCGACGACTAGTACTCGT | 5.7644 | 14.3117 | 14.3117 | 23.8424 | 38.1541 | 0.5 | -2.63 | -1.97 | 6.73431 | 5.7644 | 7.48742 | 6.7614 | 6.7614 | 12.26947 | 0.048 | 0.0291 | 2.31E-04 | 1 |
| 45 | CTGGTCGACGACTAGTACTCGT | 4.5396 | 12.1094 | 12.1094 | 25.2933 | 37.4027 | 0.6667 | -2.83 | -1.97 | 6.3665 | 4.5396 | 6.85935 | 5.946 | 5.946 | 14.3638 | 0.0748 | 0.321 | 2.97E-04 | 1 |
| 46 | CAATGCGACGACTAGTACTCGT | 5.7417 | 14.2284 | 14.2284 | 23.8417 | 38.0701 | 0.5 | -2.65 | -1.97 | 6.75768 | 5.7417 | 7.41337 | 6.7454 | 6.7454 | 13.9791 | 0.0589 | 0.0426 | 3.78E-04 | 1 |
| 47 | TGTCAGGACGACTAGTACTCGT | 0.9978 | 11.5619 | 1.9956 | 26.7448 | 38.3067 | 0.5 | -2.89 | -2.14 | 5.7653 | 4.7023 | 3.9077 | 3.7354 | 3.7354 | 13.1393 | 0.0827 | 0.00615 | 1.09E-04 | 1 |
| 48 | GAATGGGACGACTAGTACTCGT | 5.8733 | 14.6377 | 14.6377 | 23.793 | 38.4307 | 0.5 | -2.54 | -1.97 | 6.68735 | 5.8733 | 7.59768 | 6.6182 | 6.6182 | 13.80411 | 0.0384 | 0.00312 | 9.98E-05 | 1 |
| 49 | CCGATGGACGACTAGTACTCGT | 0.9676 | 10.7226 | 2.9028 | 24.5091 | 35.2317 | 0.6667 | -3.11 | -2.09 | 6.61156 | 4.1483 | 8.85984 | 7.0788 | 4.1576 | 14.7494 | 0.118 | 0.00614 | 1.54E-05 | 2 |
| 50 | TTTGGAGACGACTAGTACTCGT | 5.6526 | 13.1422 | 13.1422 | 23.7447 | 36.8869 | 0.3333 | -2.61 | -1.97 | 6.7374 | 5.6526 | 7.2448 | 6.5075 | 6.5075 | 12.12789 | 0.0446 | 0.00327 | 1.47E-05 | 2 |
| 51 | ATTTCAGACGACTAGTACTCGT | 5.9533 | 12.898 | 12.898 | 23.5179 | 36.4159 | 0.1667 | -2.5 | -1.97 | 6.75754 | 5.9533 | 7.95771 | 6.8041 | 6.8041 | 10.95329 | 0.0125 | 0.00327 | 2.19E-05 | 2 |
| 52 | GGATGAGACGACTAGTACTCGT | 5.1021 | 12.9082 | 12.9082 | 22.9388 | 35.847 | 0.5 | -2.71 | -1.97 | 6.96659 | 5.1021 | 7.07914 | 6.2372 | 6.2372 | 13.4827 | 0.0604 | 0.00257 | 2.47E-05 | 2 |
| 53 | ATCCAGGACGACTAGTACTCGT | 5.6219 | 14.0058 | 14.0058 | 23.9712 | 37.977 | 0.5 | -2.62 | -1.97 | 6.70807 | 5.6219 | 7.11406 | 6.3308 | 6.3308 | 13.1601 | 0.0598 | 0.00261 | 7.67E-05 | 1 |
| 54 | AAGCGAGACGACTAGTACTCGT | 2.9931 | 7.32681 | 6.985 | 20.8575 | 28.18431 | 0.5 | -4.44 | -3.83 | 8.01602 | 3.12189 | 13.226 | 5.8873 | 4.8888 | 12.80197 | 0.0317 | 0.00267 | 4.02E-06 | 2 |
| 55 | CGATATGACGACTAGTACTCGT | 5.6907 | 13.1573 | 13.1573 | 24.0504 | 37.2077 | 0.3333 | -2.56 | -1.97 | 6.58465 | 5.6907 | 7.65828 | 6.7807 | 6.7807 | 12.38372 | 0.0129 | 0.00459 | 7.67E-05 | 1 |
| 56 | ATGCGAGACGACTAGTACTCGT | 2.9986 | 7.33712 | 6.9967 | 20.8592 | 28.19632 | 0.5 | -4.43 | -3.83 | 8.01837 | 3.12675 | 13.2316 | 5.8885 | 4.8901 | 12.53775 | 0.0391 | 0.00268 | 3.57E-06 | 2 |
| 57 | CTGTCCGACGACTAGTACTCGT | 5.1935 | 13.8137 | 13.8137 | 25.4272 | 39.2409 | 0.6667 | -2.75 | -1.97 | 6.2007 | 5.1935 | 6.4566 | 6.5402 | 6.5402 | 14.6106 | 0.0812 | 0.0193 | 8.53E-04 | 0 |
| 58 | ACGTAGGACGACTAGTACTCGT | 0.8311 | 7.4647 | 1.6622 | 24.8627 | 32.3274 | 0.5 | -3.76 | -2.59 | 6.4619 | 3.2568 | 9.5612 | 6.9929 | 4.2226 | 12.25592 | 0.0204 | 0.0256 | 1.53E-06 | 2 |
| 59 | ATCAGCGACGACTAGTACTCGT | 5.7174 | 14.174 | 14.174 | 23.9306 | 38.1046 | 0.5 | -2.66 | -1.97 | 6.73461 | 5.7174 | 7.33692 | 6.7175 | 6.7175 | 13.4154 | 0.0806 | 0.0417 | 7.23E-04 | 0 |
| 60 | TAGGGCGACGACTAGTACTCGT | 5.6728 | 15.0483 | 15.0483 | 24.1981 | 39.2464 | 0.6667 | -2.67 | -1.97 | 6.64 | 5.6728 | 7.1728 | 6.6789 | 6.6789 | 13.957 | 0.522 | 0.0404 | 1.37E-03 | 0 |
| 61 | GGTCACGACGACTAGTACTCGT | 0.9919 | 12.4849 | 2.9757 | 25.5703 | 38.0552 | 0.6667 | -3.1 | -2.05 | 6.1256 | 4.6743 | 2.59945 | 2.9278 | 2.9278 | 14.9301 | 0.0536 | 0.109 | 6.82E-04 | 0 |
| 62 | CGGTTTGACGACTAGTACTCGT | 5.7806 | 14.3859 | 14.3859 | 24.0853 | 38.4712 | 0.5 | -2.55 | -1.97 | 6.56106 | 5.7806 | 7.63979 | 6.7786 | 6.7786 | 14.202 | 0.165 | 0.00477 | 1.83E-03 | 0 |
| 63 | GCCATGGACGACTAGTACTCGT | 5.811 | 15.4367 | 15.4367 | 23.7027 | 39.1394 | 0.6667 | -2.59 | -1.97 | 6.72052 | 5.81108 | 7.57669 | 6.6383 | 6.6383 | 15.1272 | 0.34 | 0.326574 | 8.12E-04 | 0 |
| 64 | GCCTGTGACGACTAGTACTCGT | 5.8467 | 15.5891 | 15.5891 | 23.7986 | 39.3877 | 0.6667 | -2.54 | -1.97 | 6.67152 | 5.84675 | 7.6926 | 6.7326 | 6.7326 | 14.6724 | 0.9 | 0.00338 | 3.73E-03 | 0 |
| 65 | AAGCGAGACGACTAGTACTCGT | 2.9931 | 7.32681 | 6.985 | 20.8575 | 28.18431 | 0.5 | -4.44 | -3.83 | 8.01602 | 3.12195 | 10.0962 | 5.8873 | 4.8888 | 12.80197 | 0.0317 | 0.00267 | 4.27E-06 | 2 |
| 66 | GTGGTCGACGACTAGTACTCGT | 5.3796 | 14.3113 | 14.3113 | 25.0894 | 39.4007 | 0.6667 | -2.72 | -1.97 | 6.3232 | 5.37953 | 6.67479 | 6.5783 | 6.5783 | 14.7067 | 0.106 | 0.106 | 1.02E-03 | 0 |
| 67 | CTGCGGGACGACTAGTACTCGT | 2.9304 | 12.0936 | 7.8039 | 28.7593 | 40.8529 | 0.8333 | -3.31 | -2.79 | 4.93593 | 4.36031 | 4.80095 | 2.5213 | 2.5213 | 15.7231 | 0.647 | 0.0301 | 5.78E-05 | 1 |
| 68 | GCGCGAGACGACTAGTACTCGT | 2.9293 | 9.43611 | 8.7879 | 21.1819 | 30.61801 | 0.8333 | -4.46 | -3.83 | 7.89181 | 3.173145 | 9.82265 | 5.8456 | 4.8489 | 15.4443 | 1.09 | 0.25723 | 1.44E-04 | 1 |
| 69 | GTTTTCGACGACTAGTACTCGT | 5.7266 | 13.1929 | 13.1929 | 23.7638 | 36.9567 | 0.3333 | -2.63 | -1.97 | 6.75908 | 5.72668 | 7.58056 | 6.7755 | 6.7755 | 13.13685 | 0.00632 | 0.0288 | 2.23E-04 | 1 |
| 70 | TCAGAGGACGACTAGTACTCGT | 2.8647 | 8.3804 | 6.6033 | 22.4016 | 30.782 | 0.5 | -3.49 | -3.2 | 7.69782 | 3.52721 | 10.9106 | 6.6618 | 4.6767 | 13.05377 | 0.0178 | 0 | 1.96E-07 | 2 |
| 71 | GCGCAAGACGACTAGTACTCGT | 0.996 | 13.3247 | 2.988 | 25.8516 | 39.1763 | 0.6667 | -3.06 | -2.05 | 5.9345 | 5.10144 | 3.92231 | 4.2285 | 4.345 | 14.8165 | 1.02 | 0.1863 | 1.69E-03 | 0 |
| 72 | AAAAAGGACGACTAGTACTCGT | 5.6586 | 12.125 | 12.125 | 23.9808 | 36.1058 | 0.1667 | -2.61 | -1.97 | 6.6979 | 5.65867 | 7.12292 | 6.3303 | 6.3303 | 11.387545 | 0.00447 | 0.00225 | 6.02E-06 | 2 |
| 73 | GCTGCGGACGACTAGTACTCGT | 5.6512 | 15.9607 | 15.9607 | 23.9641 | 39.9248 | 0.8333 | -2.63 | -1.97 | 6.64507 | 5.6512 | 7.37746 | 6.5818 | 6.5818 | 16.5729 | 3.42 | 0.005009 | 6.64E-05 | 1 |
| 74 | CACCGCGACGACTAGTACTCGT | 5.5928 | 15.7836 | 15.7836 | 24.3596 | 40.1432 | 0.8333 | -2.71 | -1.97 | 6.5683 | 5.59283 | 7.08516 | 6.6937 | 6.6937 | 15.8705 | 1.97 | 0.052012 | 3.14E-03 | 0 |
| 75 | GGGGGAGACGACTAGTACTCGT | 5.6447 | 16.0973 | 16.0973 | 23.8428 | 39.9401 | 0.8333 | -2.62 | -1.97 | 6.7044 | 5.64473 | 7.16386 | 6.4852 | 6.4852 | 13.9885 | 1.55 | 0.00309 | 6.06E-04 | 0 |
| 76 | CGAATGGACGACTAGTACTCGT | 5.4204 | 13.3562 | 13.3562 | 23.974 | 37.3302 | 0.5 | -2.65 | -1.97 | 6.67119 | 5.42037 | 7.25673 | 6.4425 | 6.4425 | 14.3045 | 0.0539 | 0.00334 | 2.07E-03 | 0 |
| 77 | AACTCAGACGACTAGTACTCGT | 5.7397 | 13.3813 | 13.3813 | 23.9852 | 37.3665 | 0.3333 | -2.56 | -1.97 | 6.6252 | 5.73965 | 7.37604 | 6.5776 | 6.5776 | 11.6543 | 0.0508 | 0.00301 | 2.50E-04 | 1 |
| 78 | GGTGGCGACGACTAGTACTCGT | 5.621 | 15.8972 | 15.8972 | 24.2574 | 40.1546 | 0.8333 | -2.69 | -1.97 | 6.6121 | 5.27424 | 7.17601 | 6.6999 | 6.6999 | 16.1135 | 0.981 | 0.0384 | 2.32E-03 | 0 |
| 79 | TTTTTTGACGACTAGTACTCGT | 5.9967 | 11.9934 | 11.9934 | 23.2996 | 35.293 | 0 | -2.48 | -1.97 | 6.82751 | 5.9967 | 8.15018 | 6.8468 | 6.8468 | 7.0110244 | 0.00409 | 0.00321 | 1.55E-05 | 2 |
| 80 | AGGAAAGACGACTAGTACTCGT | 5.9347 | 13.8509 | 13.8509 | 23.6131 | 37.464 | 0.3333 | -2.51 | -1.97 | 6.72675 | 5.93464 | 7.8498 | 6.7674 | 6.7674 | 12.29427 | 0.032 | 0.00326 | 1.15E-04 | 1 |
| 81 | AATGGTGACGACTAGTACTCGT | 5.8548 | 13.6411 | 13.6411 | 23.8504 | 37.4915 | 0.3333 | -2.53 | -1.97 | 6.65319 | 5.8548 | 7.74051 | 6.7456 | 6.7456 | 12.095 | 0.0614 | 0.00376 | 1.12E-04 | 1 |
| 82 | ACTCCGGACGACTAGTACTCGT | 5.613 | 14.8862 | 14.8862 | 24.0539 | 38.9401 | 0.66666667 | -2.64 | -1.97 | 6.6149 | 5.61309 | 7.28967 | 6.567 | 6.567 | 14.6696 | 0.55 | 0.0161 | 5.27E-05 | 1 |
| 83 | CCCTCCGACGACTAGTACTCGT | 5.7928 | 16.383 | 16.383 | 23.7535 | 40.1365 | 0.83333333 | -2.61 | -1.97 | 6.75653 | 5.79283 | 7.563 | 6.7765 | 6.7765 | 15.6726 | 0.36 | 0.0267 | 4.45E-03 | 0 |
| 84 | TTGGTCGACGACTAGTACTCGT | 5.4372 | 13.4735 | 13.4735 | 25.0234 | 38.4969 | 0.5 | -2.7 | -1.97 | 6.3457 | 5.43717 | 6.72815 | 6.597 | 6.597 | 13.2088 | 0.0551 | 0.0947 | 3.94E-04 | 1 |
| 85 | TCACCAGACGACTAGTACTCGT | 5.8523 | 14.6248 | 14.6248 | 23.6881 | 38.3129 | 0.5 | -2.55 | -1.97 | 6.70552 | 5.85235 | 7.71171 | 6.7471 | 6.7471 | 12.437 | 0.472 | 0.0036 | 3.89E-04 | 1 |
| 86 | TCAATTGACGACTAGTACTCGT | 5.9266 | 12.7952 | 12.7952 | 23.3434 | 36.1386 | 0.16666667 | -2.52 | -1.97 | 6.81831 | 5.92666 | 8.0381 | 6.8415 | 6.8415 | 11.06005 | 0.507 | 0.4298 | 3.56E-05 | 2 |
| 87 | TCCGTAGACGACTAGTACTCGT | 5.1817 | 12.6738 | 12.6738 | 25.9633 | 38.6371 | 0.5 | -2.84 | -1.97 | 5.9233 | 5.18173 | 6.22835 | 6.587 | 6.587 | 11.932 | 0.406 | 0.01258 | 8.37E-05 | 1 |
| 88 | TGAGTAGACGACTAGTACTCGT | 0.998 | 6.6879 | 1.996 | 25.8404 | 32.5283 | 0.33333333 | -3.26 | -2.37 | 6.481 | 2.9665 | 7.65578 | 7.05 | 7.05 | 10.95959 | 0.0213 | 0.000917 | 5.21E-06 | 2 |
| 89 | CCTTTAGACGACTAGTACTCGT | 5.8524 | 13.6482 | 13.6482 | 23.7997 | 37.4479 | 0.33333333 | -2.56 | -1.97 | 6.6757 | 5.85237 | 7.54282 | 6.6921 | 6.6921 | 11.68309 | 0.0207 | 0.00342 | 2.24E-05 | 2 |
| 90 | CCACTCGACGACTAGTACTCGT | 5.6739 | 15.0964 | 15.0964 | 24.117 | 39.2134 | 0.66666667 | -2.65 | -1.97 | 6.6466 | 5.67393 | 7.19523 | 6.6519 | 6.6519 | 14.4631 | 0.105 | 0.0269 | 2.31E-03 | 0 |
| 91 | TTCGTGGACGACTAGTACTCGT | 1.9654 | 8.8354 | 3.9308 | 23.6264 | 32.4618 | 0.5 | -3.31 | -2.49 | 6.9312 | 3.9312 | 10.0659 | 8.1944 | 4.3994 | 13.1961 | 0.0554 | 0.00625 | 1.15E-05 | 2 |
| 92 | CTCGTGGACGACTAGTACTCGT | 1.9446 | 9.8308 | 4.8745 | 23.679 | 33.5098 | 0.66666667 | -3.32 | -2.94 | 6.9112 | 3.9272 | 9.9736 | 8.1631 | 4.3784 | 11.3591 | 0.113 | 0.00604 | 2.28E-05 | 2 |
| 93 | TCCGTGGACGACTAGTACTCGT | 1.973 | 10.055 | 4.9227 | 23.4023 | 33.4573 | 0.66666667 | -3.27 | -2.9 | 7.00298 | 4.0144 | 10.02257 | 8.174 | 4.4141 | 14.0676 | 0.426 | 0.00329 | 3.39E-05 | 2 |
| 94 | CCCGTGGACGACTAGTACTCGT | 1.9433 | 11.0213 | 5.8299 | 23.4086 | 34.4299 | 0.83333333 | -3.28 | -2.9 | 6.996 | 4.0044 | 9.93405 | 8.1347 | 4.3975 | 15.6693 | 0.566 | 0.00344 | 8.72E-05 | 1 |
| 95 | TTTGTGGACGACTAGTACTCGT | 5.7968 | 13.4339 | 13.4339 | 24.0557 | 37.4896 | 0.33333333 | -2.57 | -1.97 | 6.61514 | 5.7968 | 7.34711 | 6.5283 | 6.5283 | 12.78171 | 0.027 | 0.00283 | 5.74E-05 | 1 |
| 96 | CTTGTGGACGACTAGTACTCGT | 5.6565 | 14.0554 | 14.0554 | 24.1939 | 38.2493 | 0.33333333 | -2.61 | -1.97 | 6.57661 | 5.6565 | 7.21972 | 6.4825 | 6.4825 | 13.87805 | 0.0444 | 0.00287 | 6.21E-05 | 1 |
| 97 | TCTGTGGACGACTAGTACTCGT | 5.6975 | 14.1519 | 14.1519 | 24.1118 | 38.2637 | 0.5 | -2.61 | -1.97 | 6.60185 | 5.6975 | 7.25633 | 6.5135 | 6.5135 | 13.0592 | 0.141 | 0.00314 | 4.27E-05 | 2 |
| 98 | CCTGTGGACGACTAGTACTCGT | 5.5707 | 14.7595 | 14.7595 | 24.088 | 38.8475 | 0.66666667 | -2.65 | -1.97 | 6.60677 | 5.5707 | 7.25011 | 6.5327 | 6.5327 | 14.6692 | 0.181 | 0.00355 | 7.87E-05 | 1 |
| 99 | TCCGAGGACGACTAGTAGACGT | 5.5183 | 14.58571 | 14.58571 | 21.62783 | 36.21354 | 0.666666667 | -3.14 | -2.24 | 6.713323 | 5.5183 | 10.80279 | 6.223087 | 4.233179 | 14.10628 | 0.652 | 0.023176 | 1.60E-04 | 1 |
| 100 | TCCGAGGACGACTAGTAGTCGA | 5.788345 | 15.40754 | 15.40754 | 14.87727 | 30.28481 | 0.666666667 | -6 | -5.54 | 10.02303 | 5.788345 | 10.9335 | 4.0683 | 4.0683 | 14.32133 | 1.19 | 0.03584 | 9.90E-06 | 2 |
| 101 | TCCGAGGACGACTAGTACTCCT | 2.879442 | 8.941114 | 7.639426 | 23.80485 | 32.745964 | 0.666666667 | -3.47 | -3.24 | 6.276071 | 3.372722 | 10.67561 | 6.679201 | 4.787199 | 12.65635 | 0.0182 | 0.00731 | 4.43E-06 | 2 |
| 102 | TCCGAGGACGACTAGTACTGCT | 2.840913 | 10.84491 | 7.527513 | 27.56285 | 38.40776 | 0.666666667 | -2.76 | -1.94 | 4.725945 | 4.17818 | 4.785095 | 6.006305 | 4.03581 | 13.99264 | 0.0702 | 0.0235 | 5.60E-05 | 1 |
| 103 | TCCGAGGACGACTAGTAGTCGT | 5.891285 | 14.70431 | 14.70431 | 11.641 | 26.34531 | 0.666666667 | -6.17 | -5.52 | 11.16079 | 5.891285 | 12.21155 | 3.533495 | 3.53495 | 14.10297 | 0.799 | 0.04232 | 3.58E-06 | 2 |
| 104 | TCAGAGGACGACTAGTACTCAT | 2.995837 | 8.125212 | 7.98803 | 21.04754 | 29.172752 | 0.5 | -4.12 | -4.08 | 6.975182 | 3.04606 | 12.69556 | 6.99791 | 5 | 12.51031 | 0.000599 | 0 | 2.94E-07 | 2 |
| 105 | TCAGAGGACGACTAGGACTCAT | 2.933265 | 8.147613 | 6.820433 | 29.42421 | 37.571823 | 0.5 | -2.23 | -1.67 | 3.835251 | 3.422987 | 7.484877 | 8.572962 | 4.788439 | 13.11525 | 0.0106 | 0 | 1.27E-07 | 2 |
| 106 | TCAGAGGACGACTAAGACTCAT | 2.909531 | 8.490459 | 6.759135 | 30.25512 | 38.745579 | 0.5 | -1.93 | -1.41 | 3.222628 | 3.546919 | 5.648055 | 10.0007 | 10.0007 | 13.08639 | 0.0126 | 0 | 2.34E-05 | 2 |
| 107 | TCAGAGGACGACTAAGACTCTT | 1.93455 | 7.37372 | 4.815636 | 28.46981 | 35.84353 | 0.5 | -2.25 | -1.54 | 4.084402 | 3.004354 | 6.969034 | 9.921567 | 9.921567 | 13.18081 | 0.0137 | 0 | 4.81E-06 | 2 |
| 108 | TCAGAGGACGACTAAGAATCTG | 0.973203 | 7.34716 | 1.946406 | 29.93025 | 37.27741 | 0.5 | -1.92 | -1.4 | 3.56635 | 2.975867 | 6.342376 | 13.22028 | 13.22028 | 13.13856 | 0.0152 | 0 | 3.53E-05 | 2 |
| 109 | TCCGAGGACGACTAGACTCGT | 1.988847 | 6.379438 | 4.968162 | 23.49499 | 29.874428 | 0.666666667 | -4.04 | -3.62 | 5.225531 | 2.503395 | 10.90848 | 6.750678 | 4.771242 | 14.10513 | 0.0865 | 0 | 2.64E-05 | 2 |
| 110 | TCCGAGGACGACTAACTCGT | 1.968325 | 9.516499 | 4.909989 | 21.24402 | 30.760519 | 0.666666667 | -3.82 | -3.37 | 5.211847 | 3.645986 | 6.134083 | 7.401391 | 7.401391 | 14.10325 | 0.318 | 0.000852 | 1.43E-04 | 1 |
| 111 | TCCGAGGACGACTAGTACATCGT | 4.145779 | 10.87023 | 10.87023 | 24.81254 | 35.68277 | 0.666666667 | -3.45 | -2.42 | 6.498831 | 4.145779 | 3.184942 | 6.659909 | 6.659909 | 14.09193 | 0.309 | 0.00123 | 8.14E-06 | 2 |
| 112 | TCAGAGGACGACTAGTACTGT | 3.047135 | 9.484922 | 7.503309 | 23.64649 | 33.131412 | 0.5 | -2.47 | -1.94 | 5.277815 | 3.856587 | 6.46585 | 6.798031 | 4.827131 | 12.84188 | 0.00931 | 0.000914 | 9.12E-06 | 2 |
| 113 | TCAGAGGACGACTGTACTGT | 2.58341 | 8.873721 | 6.267903 | 23.23416 | 32.107881 | 0.5 | -2.41 | -1.66 | 4.615319 | 3.641471 | 4.548542 | 5.422086 | 3.465401 | 12.86844 | 0.00809 | 0.000669 | 9.79E-06 | 2 |
| 114 | TCAGAGGACGACTAGATACTCGT | 5.604897 | 13.9429 | 13.9429 | 25.78343 | 39.72633 | 0.5 | -2.63 | -1.97 | 6.279264 | 5.604897 | 7.110981 | 7.53205 | 7.53205 | 13.08767 | 0.0816 | 0 | 3.00E-06 | 2 |

**6. References**

(1) Zhang, D. Y.; Winfree, E. Control of DNA strand displacement kinetics using toehold exchange. *J. Am. Chem. Soc.* **2009,** *131* (47), 17303.

(2) Zadeh, J. N.; Steenberg, C. D.; Bois, J. S.; Wolfe, B. R.; Pierce, M. B.; Khan, A. R.; Dirks, R. M.; Pierce, N. A. NUPACK: Analysis and design of nucleic acid systems. *J. Comput. Chem.* **2011,** *32* (1), 170.

(3) QUINLAN, J. R. Induction of Decision Trees. *Machine Learning* **1986,** *1*, 81.

(4) Tolles, J.; Meurer, W. J. Logistic Regression Relating Patient Characteristics to Outcomes. *JAMA.* **2016,** *316* (5), 533.

(5) Valkenborg, D.; Rousseau, A. J.; Geubbelmans, M.; Burzykowski, T. Support vector machines. *Am. J. Orthod.* **2023,** *164* (5), 754.

(6) Yang, A.; Zhang, W.; Wang, J.; Yang, K.; Han, Y.; Zhang, L. Review on the Application of Machine Learning Algorithms in the Sequence Data Mining of DNA. *Front. Bioeng. Biotechnol.* **2020,** *8*, 1032.
